# Pan-cancer surveys indicate cell cycle-related roles of primate-specific genes in tumors and embryonic cerebrum

**DOI:** 10.1186/s13059-022-02821-9

**Published:** 2022-12-06

**Authors:** Chenyu Ma, Chunyan Li, Huijing Ma, Daqi Yu, Yufei Zhang, Dan Zhang, Tianhan Su, Jianmin Wu, Xiaoyue Wang, Li Zhang, Chun-Long Chen, Yong E. Zhang

**Affiliations:** 1 Key Laboratory of Zoological Systematics and Evolution & State Key Laboratory of Integrated Management of Pest Insects and Rodents, Institute of Zoology, Chinese Academy of Sciences, Beijing, 100101 China; 2 University of Chinese Academy of Sciences, Beijing, 100049 China; 3 School of Engineering Medicine, Key Laboratory of Big Data-Based Precision Medicine (Ministry of Industry and Information Technology), and Beijing Advanced Innovation Center for Big Data-Based Precision Medicine, Beihang University, Beijing, 100191 China; 4 School of Life Sciences, Nanjing University, Nanjing, 210093 China; 5 Key Laboratory of Carcinogenesis and Translational Research (Ministry of Education/Beijing), Center for Cancer Bioinformatics, Peking University Cancer Hospital & Institute, Beijing, 100142 China; 6 State Key Laboratory of Medical Molecular Biology, Department of Biochemistry and Molecular Biology, Institute of Basic Medical Sciences Chinese Academy of Medical Sciences, School of Basic Medicine Peking Union Medical College, Beijing, China; 7 Chinese Institute for Brain Research, Beijing, 102206 China; 8 Institut Curie, Université PSL, Sorbonne Université, CNRS UMR3244, Dynamics of Genetic Information, 75005 Paris, France; 9 CAS Center for Excellence in Animal Evolution and Genetics, Chinese Academy of Sciences, Kunming, 650223 China

**Keywords:** Molecular atavism, Antagonistic pleiotropy, Primate-specific genes, Cancer evolution, Brain evolution, Cell cycle, Gene duplication, *DDX11*

## Abstract

**Background:**

Despite having been extensively studied, it remains largely unclear why humans bear a particularly high risk of cancer. The antagonistic pleiotropy hypothesis predicts that primate-specific genes (PSGs) tend to promote tumorigenesis, while the molecular atavism hypothesis predicts that PSGs involved in tumors may represent recently derived duplicates of unicellular genes. However, these predictions have not been tested.

**Results:**

By taking advantage of pan-cancer genomic data, we find the upregulation of PSGs across 13 cancer types, which is facilitated by copy-number gain and promoter hypomethylation. Meta-analyses indicate that upregulated PSGs (uPSGs) tend to promote tumorigenesis and to play cell cycle-related roles. The cell cycle-related uPSGs predominantly represent derived duplicates of unicellular genes. We prioritize 15 uPSGs and perform an in-depth analysis of one unicellular gene-derived duplicate involved in the cell cycle, *DDX11*. Genome-wide screening data and knockdown experiments demonstrate that *DDX11* is broadly essential across cancer cell lines. Importantly, non-neutral amino acid substitution patterns and increased expression indicate that *DDX11* has been under positive selection. Finally, we find that cell cycle-related uPSGs are also preferentially upregulated in the highly proliferative embryonic cerebrum.

**Conclusions:**

Consistent with the predictions of the atavism and antagonistic pleiotropy hypotheses, primate-specific genes, especially those PSGs derived from cell cycle-related genes that emerged in unicellular ancestors, contribute to the early proliferation of the human cerebrum at the cost of hitchhiking by similarly highly proliferative cancer cells.

**Supplementary Information:**

The online version contains supplementary material available at 10.1186/s13059-022-02821-9.

## Background

Darwinian medicine has long been believed to provide insights into cancer diagnosis or treatment [1–4]. Specifically, concepts and strategies related to microevolution have been extensively used to model tumorigenesis, where inference of clonal history and identification of driver mutations are routinely performed within evolutionary frameworks [5–9]. In addition, macroevolutionary studies have also indicated that social changes between us and our ancestors could increase tumor risk and the genetic specificity of long-lived animals may offer clues for human cancer therapies [1, 10–13].

Micro- and macroevolutionary practices could be combined to better understand cancer as in studies of molecular atavism and antagonistic pleiotropy. On the one hand, it was hypothesized decades ago that cancer cells are reminiscent of unicellular ancestors and oncogenesis emerges by reversing phylogeny [14, 15]. Nonetheless, this atavistic view was formally formulated more recently [16–18]. Phylostratigraphic or gene age analyses provide abundant supporting evidence: (1) cancer-related genes often emerge in unicellular (UC) ancestors or early metazoan (EM) ancestors [19–22]; (2) UC genes tend to be upregulated in tumors, while EM genes are often downregulated [23, 24]. Therefore, atavism is increasingly accepted as a theoretical framework to understand cancer [4, 25–27] and adaptive mutability enabled by the ancient memory is even proposed as a target in tumor therapy [22, 28]. On the other hand, natural selection may maximize the fitness in youth at the cost of promoting diseases of aging [3, 29, 30]. Antagonism could be most intense for recent genetic changes due to lack of time resolving the negative pleiotropy [31, 32]. This hypothesis has rarely been tested at the genome-wide level with few exceptions, e.g., one study has shown that human-specific enhancers underlie the cost of aging diseases including cancer [33].

Antagonistic pleiotropy predicts that recently originated new genes (novel gene loci emerging in recent evolution) [34, 35] should often contribute to tumor. Considering atavism and the fact that the majority of new genes are generated by DNA- or RNA-level duplication [36, 37], the link between tumors and new genes could be more pronounced for new genes as derived duplicates of UC genes. That is, primate-specific genes (PSGs, including human-specific genes) harbored by the human genome should fit these patterns. Studies including our own have already indicated tumor-promoting roles for a handful of PSGs, in addition to their normal function in fetal brain development or spermatogenesis [38–43].

Therefore, we analyzed whether PSGs, especially UC gene-derived PSGs, promote tumors and what normal functions of PSGs are hitchhiked by tumors, especially considering that most PSGs are poorly characterized [44, 45]. Specifically, by taking advantage of data generated by The Cancer Genome Atlas (TCGA) project [46], we demonstrated a pan-cancer global upregulation of PSGs, which was contributed by copy number gains and promoter hypomethylation. By integrating clinical data and cell line screening data, we showed that upregulated PSGs tend to facilitate tumors possibly due to their roles in the cell cycle. Furthermore, the majority of these cell cycle-related PSGs are derived duplicates of UC genes. For one particularly strong case, *DDX11*, we corroborated its essentiality in cancer cells via knockdown assays and revealed its fast evolution shaped by natural selection. We finally showed that upregulated PSGs associated with the cell cycle, including *DDX11,* are also biasedly expressed during embryonic cerebral development, which involves extensive cell proliferation, suggesting that similarly highly proliferative tumor recapitulated part of the cell cycle program that normally acts during this critical stage.

## Results

### Compilation of the gene age dataset and cancer omics datasets

**Fig. 1:**
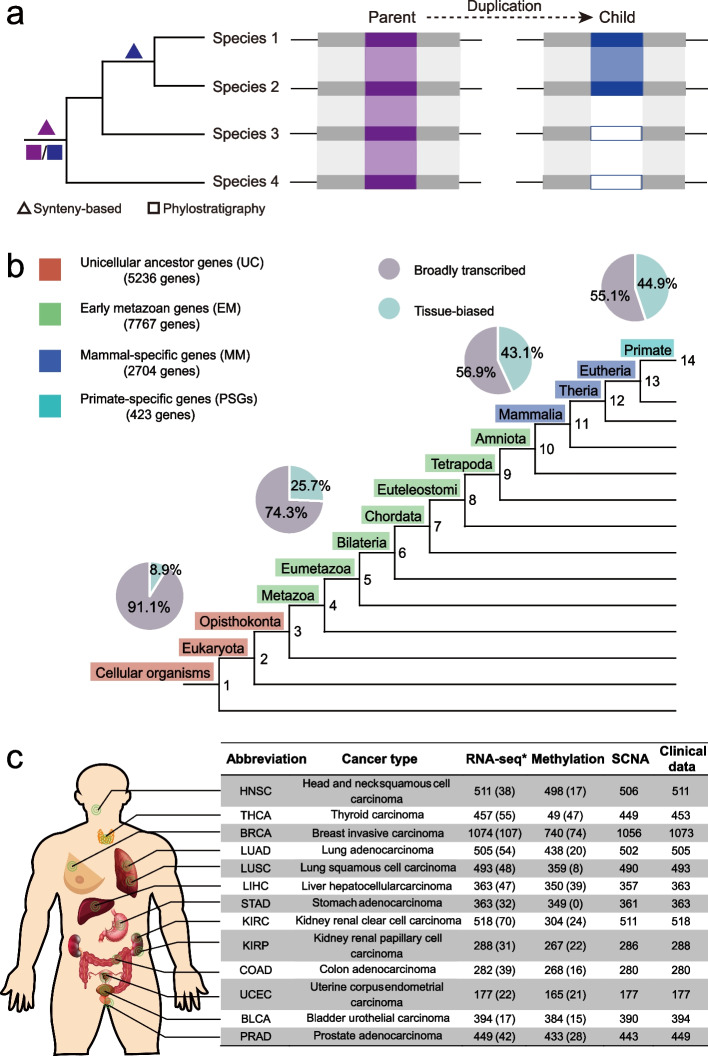
Data compilation. **a** Schema of two strategies for dating gene ages. A phylogenetic tree covering four species is shown and the age inferences are marked along the tree. The purple and blue boxes mark the parental and child copies, respectively. The gray boxes indicate the genes around the focal gene while the white boxes indicate the absence of the child gene. The shaded region represents the syntenic alignment. **b** Age assignment with pie charts showing the proportions of broadly transcribed and tissue-biased genes. **c** Count of the pan-cancer TCGA datasets used in this study. Only RNA-sequencing transcriptome datasets (marked with “*”) were reprocessed, while all other datasets were directly retrieved from previous publications or dedicated websites (“Methods”). Numbers refer to the counts of tumor samples, while numbers in parentheses refer to the counts of normal controls. SCNA refers to somatic copy number alteration

To analyze the roles of PSGs in tumors, we first compiled a genome-wide gene age dataset spanning the most ancient UC genes to the youngest PSGs. Specifically, two strategies for dating gene origination have been developed: protein-family-based methods, also known as phylostratigraphy [22, 23, 47–49], and synteny-based methods [36, 37, 40, 50–53]. The former assigns the age of the founder gene to all homologous genes of the same gene family and thus does not differentiate between the child duplicates and the parental copy (Fig. 1a). In contrast, the latter distinguishes different duplicates on the basis of synteny (gene order) and parsimoniously infers the corresponding age. Thus, for genes harbored by the human genome, PSGs identified by the former strategy indicate that these genes form primate-specific protein families (absent in non-primate species), while those genes identified with the latter strategy represent primate-specific loci.

We compiled a single gene age dataset by merging these two dating strategies given their complementary nature (“Methods”). Since synteny degenerates faster than protein sequence during evolution, the synteny-based pipeline has been applied to date human genes within vertebrate evolution [40], while protein-level homology is detectable over a much longer period, thus, phylostratigraphy has been implemented to date ancient genes [22, 23]. We thus used the age data of groups 9–14 after vertebrate split (Fig. 1b; “Methods”) generated via our previous synteny-based pipeline [40]. Genes predating the vertebrate split were classified into eight groups in a relatively new phylostratigraphy dataset [23]. We further divided all groups into 5236 UC genes, 7767 EM genes, 2704 mammal-specific (MM) genes, and 423 PSGs (Fig. 1b, Additional file 1: Fig. S1a; Additional file 2: Table S1) and considered that UC and EM genes could serve as two controls in subsequent analyses given their respective roles in tumor promotion or repression [19–24].

Notably, the dataset of PSGs shows the well-known transcriptional and evolutionary features of young genes, i.e., narrow expression [35, 54, 55] and fast evolution [56, 57]. First, almost half (190/423) of the PSGs showed tissue-biased expression (e.g., testis bias, Additional file 1: Fig. S1b; Methods), while this proportion declined in the three older age groups (43%-9%, Fig. 1b; Additional file 2: Table S1). Actually, due to pervasive incomplete duplication or relocation [58, 59], duplicate PSGs generally show narrower expression than their corresponding parental copies (Additional file 1: Fig. S1c). Second, duplicate PSGs tend to evolve faster than their older counterparts (Additional file 1: Fig. S1d) driven by positive selection or relaxation of functional constraint [56, 57, 60].

We subsequently retrieved cancer omics data. Among the 33 cancer types covered by TCGA, we focused on 13 cancer types with at least 15 normal control samples (Fig. 1c).

### PSGs are generally upregulated in tumors

**Fig. 2:**
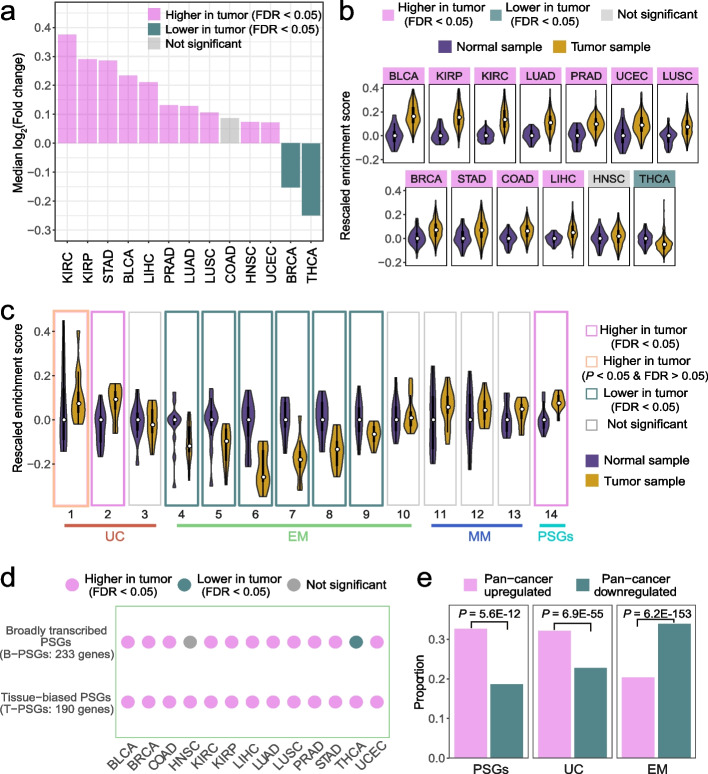
Upregulation of PSGs in tumors. **a** Distribution of the median log_2_(Fold change) of 423 PSGs across tumors. Tumor types are sorted by decreasing values. **b** Distribution of the ssGSEA enrichment scores of PSGs across tumor types. For each violin plot, the bar, the line, and the curve indicate the interquartile range, the median, and the probability density of the data, respectively. The median score of PSGs in normal samples was set as 0, i.e., the normal median was subtracted from its counterpart in tumor samples. Tumors are sorted by decreasing scores. **c** Violin plots showing distributions of enrichment scores (median across tumors) of genes within 14 age groups. For each group (defined as in Fig. 1b), the median enrichment score was calculated separately for normal and tumor samples within each tumor type. Similar to Panel b, the median values in normal samples were set as 0. **d** Enrichment score-based expression patterns of broadly transcribed PSGs and tissue-biased PSGs across tumors. **e** Proportion comparison between pan-cancer upregulated and downregulated genes. A one-sided Wilcoxon signed-rank test was used for panels **a**–**d** and a one-sided binomial test was used for panel **e**. Multiple testing correction was performed via false discovery rate (FDR)

We identified the pan-cancer upregulation patterns of PSGs by analyzing RNA-sequencing (RNA-seq) data with paralogous similarity taken into account (“Methods”). We found that the median log_2_ transformed fold changes of PSGs between tumor and normal samples were significantly higher than 0 in 10 of 13 tumor types (Fig. 2a, Additional file 2: Table S1). Moreover, we implemented the single sample gene set enrichment analysis framework (ssGSEA, “Methods”), which could detect gene sets with moderate but robust signals of expression change [61, 62]. We found that the ssGSEA enrichment scores of PSGs in tumor samples were again significantly higher than the normal ones for 11 out of 13 tumors (Fig. 2b, Additional file 2: Table S2).

**Fig. 3:**
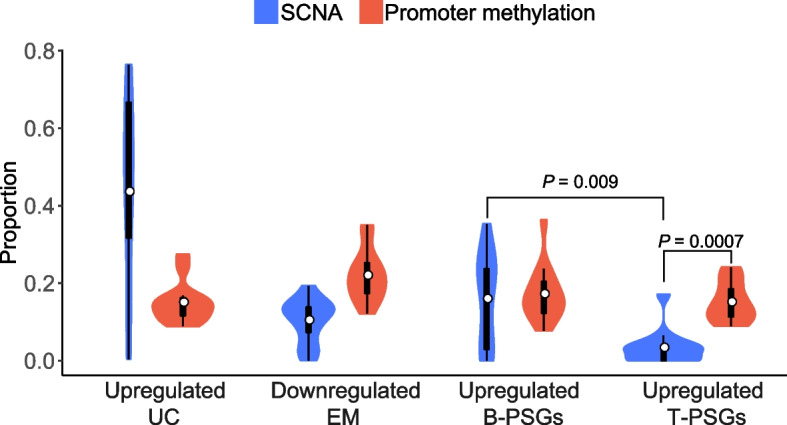
Pan-cancer proportion distribution of genes whose expression levels are strongly correlated with SCNA or promoter methylation. The Wilcoxon signed-rank test was used to measure the significance

The robustness of the upregulation pattern is supported by the following observations: (1) the overall pattern is reproducible across two methods where the fold changes are correlated with the enrichment scores (Spearman *ρ* = 0.61, *P* = 0.03); (2) our analyses recapitulated the previous studies on atavism [20–23], in that UC genes (especially those in groups 1 and 2) are upregulated, EM genes are downregulated and MM genes do not show significant patterns across tumors (Fig. 2c); (3) despite their distinct regulatory contexts, both broadly transcribed PSGs (B-PSGs) and tissue-biased PSGs (T-PSGs) are upregulated across tumors (Fig. 2d); and (4) the pattern is robust with only high-purity (no contamination of neighboring immune or stromal cells) tumor samples (Additional file 1: Figs S2a-c, Methods).

Given the overall higher expression of PSGs across tumors, we expect that PSGs should be enriched with pan-cancer upregulated genes. To test this hypothesis, we defined such genes as those that were upregulated in at least three times more cancer types than they were downregulated in. Analogously, we defined the pan-cancer downregulated genes. Consistent with our expectation, 138 out of 423 (32.6%) PSGs and 79 (18.7%) PSGs showed a pan-cancer up- and downregulation pattern, respectively (Fig. 2e). UC genes showed a similar trend (32.1% *vs.* 22.7%), while EM genes showed the opposite trend (20.3% *vs.* 33.9%). Similar results were observed with a more stringent cutoff (Additional file 1: Fig. S2d, Methods).

Collectively, PSGs tended to show higher expression in most tumor types compared to normal samples.

### Amplification and promoter hypomethylation contribute to the upregulation of PSGs

PSGs’ higher tumor expression might reflect their lower expression in normal samples, and thus they could therefore be more easily upregulated in tumors. Indeed, both B- and T-PSGs upregulated in tumors tended to have lower expression in normal controls compared to those downregulated in tumors (Additional file 1: Fig. S3a). However, UC and EM genes showed an analogous pattern. Moreover, although B-PSGs are more highly expressed than T-PSGs in normal samples, both showed a similar extent of upregulation in tumors (Additional file 1: Figs. S3a-b). Therefore, the upregulation of PSGs in tumors cannot be solely attributed to their lower expression level in normal samples.

Since gene expression in tumors is shaped by a myriad of factors including somatic copy number alterations (SCNAs), DNA methylation, or histone modifications [24, 63, 64], we wondered which factors underlie the pan-cancer upregulation of PSGs. Considering that SCNA and promoter methylation are among the most often studied factors in gene deregulation in tumors [65, 66] and that these two types of data are available for the majority of TCGA samples (Fig. 1c), we analyzed their contribution by calculating how strong gene expression is correlated with copy number and promoter methylation, respectively.

We detected varying correlation intensity across gene age groups (Fig. 3): (1) consistent with [24], SCNA (and more specifically, amplification) is one major factor underlying the upregulation of UC genes with a median of 47.9% genes showing a strong correlation (Spearman *ρ* > 0.3, “Methods”) between expression level and copy number; (2) the extent of correlation between expression and SCNA is relatively moderate for EM genes, T-PSGs and B-PSGs with the proportions ranging between 2.9% and 20.0% and the pattern for methylation is similar (13.8%-21.5%); (3) amplification contributes much more toward upregulation of B-PSGs than to that of T-PSGs (20.0% *vs.* 2.9%); and (4) compared to amplification, the decrease of methylation or hypomethylation is a stronger factor for the upregulation of T-PSGs (2.9% *vs.* 13.8%, respectively), which echoes the significance of methylation in regulating tissue-biased oncogenes [66]. We obtained similar results with a more stringent cutoff (*ρ* > 0.4, Additional file 1: Fig. S3c).

Taken together, the correlation analyses indicate that the upregulation of PSGs is contributed by amplification and promoter hypomethylation.

### Pan-cancer upregulated PSGs (uPSGs) tend to promote tumors and to play cell cycle-related roles

We next analyzed whether and how tumors benefit from upregulated PSGs by integrating survival data, pathway enrichment data, and gene essentiality data in cancer cell lines (“Methods”). To identify more important genes, we focused only on the pan-cancer up- or downregulated genes (Fig. 2e) including upregulated UC genes (uUC genes), downregulated EM genes (dEM genes), and uPSGs.

**Fig. 4:**
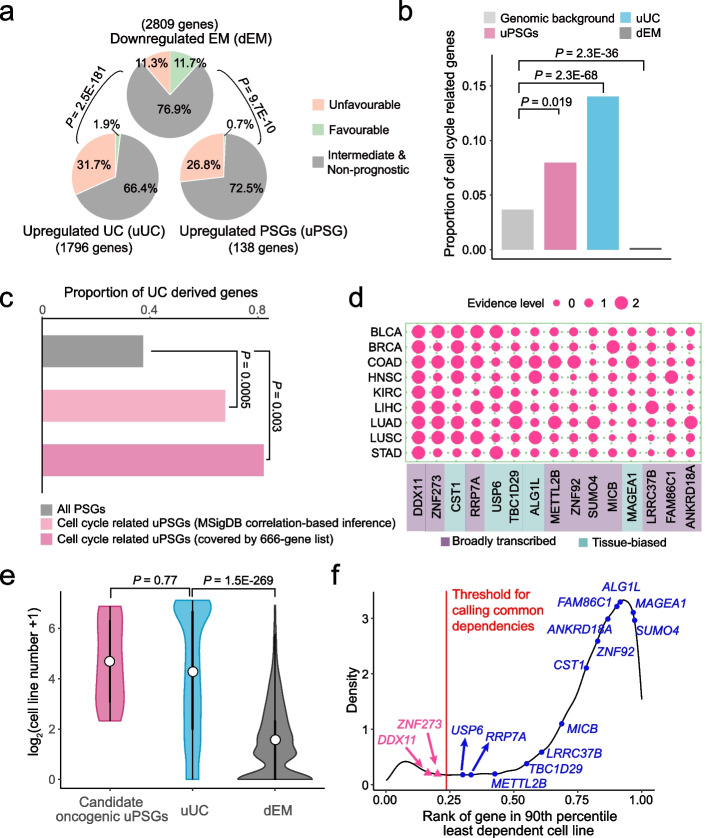
Pan-cancer upregulated PSGs (uPSGs) tend to promote tumors. **a** Higher expression of uPSGs or uUC genes more often leads to unfavorable survival compared to that of dEM genes. **b** Proportion of genes covered by the 666-gene list. **c** Proportion of PSGs dated as UC genes under the framework of phylostratigraphy. **d** A heatmap of 15 uPSGs essential for at least one cancer type. The evidence level codes the significance of one PSG for one cancer type where the small, intermediate and large circles indicate cases without upregulation and essentiality, cases with either upregulation or essentiality and cases with both signals, respectively. Expression bias is labeled. **e** Violin plots of the numbers of cell lines. For each gene, we counted how many cell lines depended on this gene and generated the plot. **f** Density distribution of gene common dependency ranks. The *X*-axis shows a relative rank value summarized across all cell lines, which indicates the overall essentiality of a gene relative to other genes. Genes with smaller values tend to be broadly essential and the common dependency cutoff was marked as a red line. Statistical tests match the context: for panel **a**, we used chi-square test; for panels **b** and **c**, binomial test was implemented; for panel **e**, we performed Wilcoxon rank-sum test

First, similar to uUC genes, uPSGs tend to promote tumors as revealed by survival data analyses (Additional file 2: Table S3; Additional file 3; Methods). Specifically, we identified genes with their expression levels significantly (false discovery rate or FDR < 0.05) correlated with the progression-free interval (PFI) of patients. We then divided genes into a favorable group, unfavorable group, intermediate group, and non-prognostic group on the basis of the number of cancer types for which their higher expression was correlated with longer or shorter PFI. The results corroborated the tumor-promoting effect of uUC genes and the tumor-inhibiting effects of dEM genes [20, 22, 23]. That is, the stronger expression of 31.7% and 1.9% of uUC genes was associated with unfavorable and favorable clinical outcomes, respectively (Fig. 4a). In contrast, the corresponding proportion becomes 11.3% and 11.7% for dEM genes, respectively. We further found that uPSGs were more similar to uUC genes in that higher expression levels of uPSGs were associated with shorter (26.8%) and longer (0.7%) PFIs, respectively. One such example is *TBC1D29* (FDR = 0.016, Additional file 1: Fig. S4a). In addition to this FDR-based analysis, we obtained similar patterns with the top 1500 genes showing the highest correlation with PFI (Additional file 1: Fig. S4b). *DDX11* serves as an example where its rank percentile reaches 0.6% (the 98th gene out of 16283 genes) despite its FDR of 0.18 (Additional file 1: Fig. S4c).

Second, functional analyses indicate that uUC genes and uPSGs tend to be involved in cell cycle. Since PSGs are generally uncharacterized [44, 45], we could not perform the conventional functional enrichment analyses. Therefore, we mapped each gene to MSigDB annotated biological processes [67] on the basis of the fact that the expressions of genes involved in similar processes tend to be correlated [68]. As predicted by the atavism hypothesis [16–18], cell proliferation or metabolic hallmarks are often enriched in uUC genes while development or signaling hallmarks are overrepresented in dEM genes (Additional file 1: Fig. S4d-e; Methods). As for uPSGs, they were overrepresented in cell proliferation hallmarks, especially in three cell cycle-related processes: mitotic spindle, G2/M checkpoint, and E2F targets (Additional file 1: Fig. S4d-e). That is, 46 uPSGs may be involved in cell cycle (Additional file 2: Table S4). In addition to the cancer-oriented annotation system, *i.e*, MSigDB, we additionally used the general annotation system of DAVID [69] and confirmed the excess of cell cycle-related uPSGs (Additional file 1: Fig. S4f; Additional file 2: Table S5; Methods).

Since correlation-based inference causes false positives or false negatives, we directly tested the enrichment of cell cycle functionality with a curated cell cycle gene list [70], which includes 666 genes involved in cell cycle progression, DNA replication or repair (“Methods”). We again found the overrepresentation of cell cycle genes in uPSGs or uUC genes and depletion of these genes in dEM genes, respectively (Fig. 4b). For example, 11 uPSGs play cell cycle-related roles (Additional file 2: Table S4), which is overrepresented compared to the genomic proportion (binomial test *P* = 0.019).

Given the atavism hypothesis, the shared excess of cell cycle-related genes in both uUC genes and uPSGs indicates the reactivation of dormant ancient memory. Thus, we expect that uPSGs potentially involved in the cell cycle should mainly represent derived duplicates of UC genes. Consistently, 67.6–81.8% of cell cycle-related uPSGs have been dated as UC genes in the protein-family-level age inference of phylostratigraphy, which is significantly higher than the overall background of PSGs (37.1%, *P* ≤ 0.003, Fig. 4c; Additional file 2: Table S4, Methods).

Third, by reprocessing CRISPR/Cas9 screening data [71] with consideration of sequence similarity (“Methods”), we prioritized 15 uPSGs that are essential for the proliferation of cancer cell lines. Specifically, given the heterogeneity within each cancer type, we focused on 9 out of 13 cancer types for which at least 5 different cell lines were screened (Additional file 1: Fig. S4g). Among the 55 uPSGs with unique single guide RNAs (sgRNAs), 10 B-PSGs and 5 T-PSGs showed dependency for at least three cell lines in one cancer type (Fig. 4d, Additional file 1: Fig. S4h; Methods). The importance of these 15 PSGs was demonstrated by the median number (23) of cell lines that were dependent on these genes. This number was analogous to that of uUC genes (20), which is subsequently higher than the number of downregulated EM genes (1, Fig. 4e). Certainly, the breadth of essentiality was uneven; some genes, such as the top two genes including *DDX11* (also named *ChlR1*) and *ZNF273*, showed a common dependency [72], whereas other genes, such as *TBC1D29*, showed relatively narrower essentiality (Fig. 4f). Notably, both *DDX11* and *ZNF273* were identified by the MSigDB based correlation method and the 666-gene list to play cell cycle-related roles, again suggesting the importance of the cell cycle program in tumors.

Altogether, UC genes-derived uPSGs potentially related with the cell cycle are more likely to be recruited into tumors.

### *DDX11* plays pan-cancer cell cycle-related roles and evolves under positive selection

**Fig. 5:**
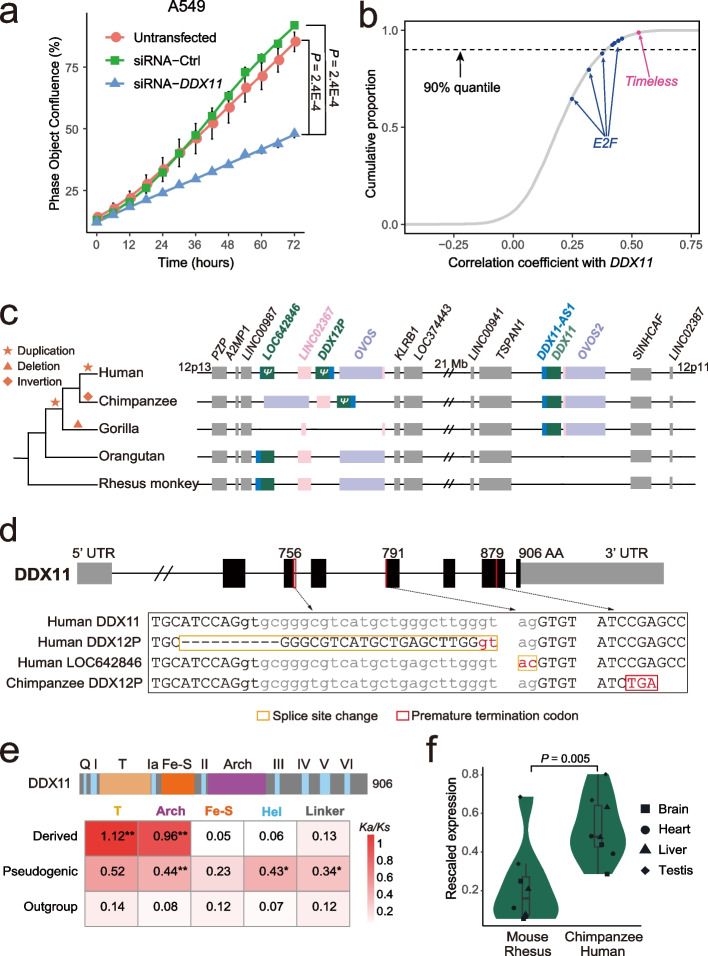
Functional and evolutionary analyses of *DDX11*. **a** Time course cell viability assay in A549 cells by Incucyte S3 live-cell analysis. Error bars indicate the standard error of the mean (SEM) calculated based on six biological replicates. The *P*-value was determined by Wilcoxon signed-rank test. **b** Genome-wide Spearman correlation coefficient distribution of *DDX11*. Eight *E2F* members and *Timeless* are marked with blue and pink dots, respectively. The dashed line denotes the 90% quantile. **c** Syntenic view across five primates. Duplicated genes are color-coded. The sizes of blocks are roughly proportional to the lengths of genes. **d** Loss-of-function (LoF) mutations accumulated in pseudogenic *DDX11* homologs. Coding exons are shown as thicker black boxes while untranslated regions are shown as thinner gray boxes. Introns are shown as connecting lines while LoF mutations are marked as red lines with the codon positions labeled. The bottom alignment shows the specific sequences flanking each LoF mutation. **e ***K*_a_*/K*_s_ distribution across five types of functional regions. Black stars indicate significantly different rates relative to the outgroup sequences (**: *P* < 0.01; *: *P* < 0.05). Small motifs are merged and labeled as helicase (Hel) motifs. Fe-S refers to an iron–sulfur cluster involved in catalysis. **f** Expression profile of *DDX11* across species. To make the expression intensity comparable across species, we normalized the raw expression values (“Methods”)

We subsequently focused on *DDX11*, which promotes tumors across all cancer types (Fig. 4d). *DDX11* has been known to be implicated in several tumors and proposed as a candidate oncogene [73–75]. We corroborated its pan-cancer role with two lines of data. First, the upregulation of *DDX11* across cancer types reached a median fold of 240%, which was higher than that of 97.4% of genes (Additional file 1: Fig. S5a). The expression of *DDX11* was correlated with copy number changes and promoter methylation changes (Additional file 1: Fig. S5b), which is in line with the general mode of B-PSGs (Fig. 3). Second, we knocked down *DDX11*’s expression in lung cancer (A549) and colon cancer (HCT116) cell lines, respectively (Additional file 1: Fig. S5c). Consistent with the genome-wide screening data (Fig. 4d), *DDX11* knockdown led to a significant decrease in proliferation in both cell lines (Fig. 5a, Additional file 1: Fig. S5d).

We inferred how *DDX11* exerts its pan-cancer role given the previous studies based on individual cell lines or individual cancer types: (1) *DDX11* is involved in DNA replication or repair, sister chromatid cohesion, and spindle assembly [75–77]; (2) it is regulated by the aforementioned *E2F* family [74, 78]; and (3) it interacts with the replication fork complex protection factor, i.e., *Timeless* [79, 80], which is also regulated by *E2F* [81]. We thus predicted that the interaction between *DDX11* and *E2F* or *Timeless* could be generalized across cancers and found two consistent patterns. First, among all eight *E2F* members, five showed a positive correlation (Spearman *ρ* > 0.4) with *DDX11*, which was higher than 90% of genes (Fig. 5b). Compared to *E2F*, *Timeless* showed an even higher correlation with *DDX11* (*ρ* = 0.526). Second, functional genomic data showed that the promoters of *DDX11* and *Timeless* were constantly bound by E2F across all eight samples covering three cancer cell lines (Additional file 1: Fig. S5e), while only 3.5% of genes showed the same pattern (Additional file 1: Fig. S5f). Thus, under the coordination of *E2F*, *DDX11* interact with *Timeless* to support the tumor cell cycle.

Similar to the majority of cell cycle-related uPSGs (Fig. 4c, Additional file 2: Table S4), *DDX11* represents a UC gene-derived duplicate that emerged in recent evolution. Specifically, synteny data across phylogenetically representative primates reveal a dynamic picture (Fig. 5c): (1) human 12p13 encodes two paralogs (*LOC642846*, *DDX12P*) separated by *LINC02367*, while the counterpart loci in other primates encodes only one copy; (2) human 12p11 harbors the currently annotated *DDX11* locus, which is only shared by chimpanzee/bonobo lineage (Additional file 1: Fig. S6a) and gorilla; and (3) the orthologs of human 12p13 has been subjected to deletions or inversions causing the loss of most sequences in gorilla and an antisense transcript (homologous to annotated *DDX11-AS1*) linked with *LOC642846* in human, as well as changes in gene orders in the chimpanzee/bonobo lineage (Additional file 1: Fig. S6b). Thus, the most parsimonious scenario is that *LOC642846* represents the ancestral copy, which has been subjected to two duplications, one in the common ancestor of humans/chimpanzees/gorillas, and one in recent human evolution, respectively (Fig. 5c).

The deletion in gorilla indicates that the ancestral copy is nonfunctional. Consistently, mutations disrupting splicing sites or premature termination codon were accumulated in orthologous copies of both humans and chimpanzees (Fig. 5d). The pseudogenic statuses of *LOC642846* and *DDX12P* were further supported by expression data, regulation data and previous literature: (1) both copies show significantly lower expression than *DDX11* across four normal human tissues (median 1.04 or 0.28 *vs.* 1.44, Additional file 1: Fig. S6c; Methods); (2) their promoters are less frequently bound by *E2Fs* relative to that of *DDX11* (Additional file 1: Fig. S6d); and (3) although *LOC642846* has not been reported, knockout of *DDX12P* does not impair cellular proliferation [82, 83].

As shown in Additional file 1: Fig. S1d, the derived copies often evolve faster, and we found that *DDX11* rapidly evolved and thus fit this general picture (“Methods”). Specifically, we quantified the protein evolution rate as the ratio between non-synonymous substitution rate and synonymous substitution rate (*K*_a_*/K*_s_) across all five functional regions of DDX11 (Additional file 1: Fig. S6e). The higher values suggest the relaxation of functional constraint or positive selection [60, 84]. Consistent with relaxation, the two presumably pseudogenic copies showed a higher *K*_a_*/K*_s_ than the single copy *DDX11* homologs in outgroups for all five regions with three (Arch, Hel, and linker) reaching statistical significance (Fig. 5e). In contrast, the protein evolution pattern of the dispersed *DDX11* was more compatible with positive selection since (1) it was constrained across three regions by showing similar or slightly lower *K*_a_*/K*_s_ relative to that in the outgroups and (2) significantly faster evolution was only detected in the remaining T and Arch regions, where T harbors the key motif that mediates the interaction between DDX11 and Timeless and Arch may interact with DNA [76, 79]. Consistent with positive selection rather than relaxation, *DDX11* in humans and chimpanzees showed much higher expression than its counterpart in outgroups including rhesus monkeys and mice (a median of 3-fold, Fig. 5f).

Therefore, positive selection may drive the enhanced function of *DDX11* by modifying the critical protein domains and increasing its expression, which could be hitchhiked by the highly proliferative cell cycles of cancer.

### Cell cycle-related uPSGs tend to be involved in embryonic cerebral development and are subjected to positive selection

Since tumor hitchhiking of UC genes derived cell cycle-related uPSGs (including *DDX11*) may represent antagonistic pleiotropy, we wondered which normal biological processes recruit these cell cycle-related new genes under the force of positive selection. Considering that increased proliferation of neural progenitor or stem cells drives human cerebral expansion [85–88] and that cancer cells have been proposed to be similar to neural stem cells [e.g., fast cell cycle or proliferation [89]], we hypothesized that these cell cycle-related uPSGs emerge due to the selection in driving brain expansion. To this end, we analyzed whether a rapid proliferation stage of brain development recruits an excess of PSGs and whether this stage is a hot target of selection.

**Fig. 6:**
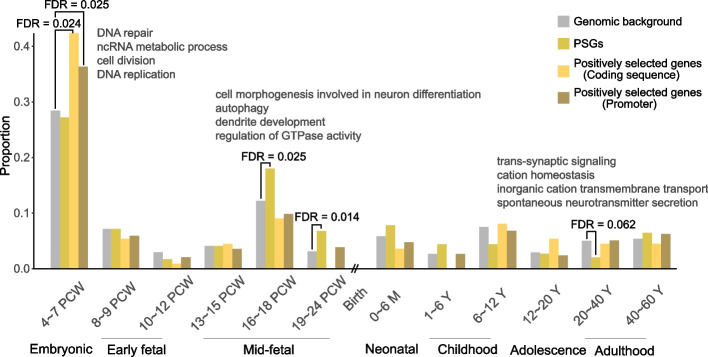
Distribution of proportions of genes highly expressed in one stage of cerebral development. Four series of proportions are shown: all genes and PSGs with biased expression in one stage, genes positively selected on coding and promoter regions. PCW, M, and Y refer to postconceptional weeks, months, and years, respectively. For each stage, we implemented binomial tests and examined whether PSGs and positively selected genes showed significant excess or depletion relative to the genomic background. Only four stages showed at least one significant (FDR < 0.1) test result and the top four GO terms for these stages are shown. For 19–24 PCW, no significant terms were identified

We reanalyzed a transcriptome dataset profiling embryonic development through the adult stages of seven organs [90]. To generate a global picture of gene expression in cerebrum (forebrain) development, we assigned genes toward one particular stage according to their biased expression (Additional file 1: Fig. S7a, Additional file 2: Table S7; Methods). We first reproduced previous observations [40, 57] (Fig. 6): (1) PSGs are disproportionately upregulated in the mid-fetal stages; (2) they are depleted in some postnatal samples in which genes involved in neuron ensheathment or synaptic transmission are disproportionately expressed. We then found that similar to the genome background the highest proportion of PSGs showed maximum expression in the embryonic stage (27.2%, Fig. 6). For this earliest stage, genes involved in the cell cycle are highly overrepresented with corresponding Gene Ontology (GO) terms (DNA replication/repair, cell division) contributing three out of the top four enriched terms (Additional file 2: Table S8). Consistently, we found the cell cycle-related uPSGs (e.g., *DDX11*) tended to be expressed in this stage regardless of whether the MSigDB correlation-based dataset (39.0%, one-sided binomial test *P* = 0.067) or the 666-gene list (80.0%, *P* = 0.0008) was used (Additional file 2: Table S4). Notably, the heightened activity of cell cycle genes was most pronounced in the cerebrum while the activity is significantly lower in other organs including cerebellum (Additional file 1: Fig. S7b).

Consistent with the positive selection signal of *DDX11* (Fig. 5e), we found that the embryonic stage was the only outlier with overrepresentation of genes with positive selection of their coding sequence or promoter regions (47 or 42.3%, 122 or 36.3%, Fig. 6; Additional file 2: Table S9; “Methods”). Consistent with the overall functional bias of embryonic genes, these 47 or 122 genes are also disproportionately related to the cell cycle (Additional file 2: Table S8).

Altogether, the cell cycle program of embryonic cerebral development seems to be rewired by recruiting PSGs and modifying preexisting genes.

## Discussion

By integrating evolutionary and functional genomic data, we found that PSGs upregulated in tumors tend to play cell cycle-related roles and that these genes mainly represent derived duplicates of UC genes. That is, we detected patterns compatible with the joint prediction of atavism and antagonistic pleiotropy. These results not only substantiate how evolutionary heritage underlies tumor risk in humans but also illuminate how our brain becomes more humanized.

On the one hand, Darwinian medicine has championed the idea that disease susceptibility is predisposed by macroevolutionary history [1, 3, 4, 91]. Accordingly, studies of atavism and antagonistic pleiotropy provide clues for understanding tumor biology [22, 28, 33]. Herein, by building upon previous efforts [19–24], our meta-analyses across 13 cancer types demonstrated that the pan-cancer upregulation of UC genes involved in basic cellular machinery tends to promote tumor (Figs. 2c and 4a, Additional file 1: Fig. S4d) and thus corroborated the atavistic hypothesis. We identified a novel pattern in which PSGs especially those UC genes derived duplicates involved in cell cycle (e.g., *DDX11*) show an analogous upregulation in tumors (Figs. 2c and 4b, c). We further prioritized 15 upregulated PSGs potentially implicated in tumor, among which, *DDX11* has already been proposed to be a target for therapeutic interventions [75]. The presence of these PSGs warns the suitability of mouse models, which echoes the limitations of mouse models of cancer due to rodent specificity [92, 93]. Note that our conclusion regarding the significance of PSGs is conservative since (1) the novel genes most likely subject to antagonism, i.e., the youngest or human-specific genes including dozens of tumor-promoting cancer-testis genes [64, 94] and genes driving brain expansion but also benefiting tumor [e.g., *NOTCH2NL* [43]] were often excluded from our analyses due to read mapping issues; (2) we used only primary tumor samples from TCGA while multiple PSGs, e.g., *POM121* [95, 96], are known to contribute to metastasis; (3) since we focused on UC genes derived uPSGs involved in cell proliferation across multiple cancer types, PSGs recruited into other hallmarks (e.g., immune invasion) or functional in specific cancer types warrant further investigation, especially considering PSGs’ biased expression in immune-related organs [Additional file 1: Fig. S1b, [40, 97]]; and (4) we herein focused on PSGs upregulated in tumors, but it is possible that the down-regulation of some PSGs might also contribute to tumor.

On the other hand, our analyses indicate that both embryonic and fetal developments of the cerebrum have been rewired during primate evolution. The classical radial unit hypothesis states that cortex size is determined by the number of earliest neural stem cells (neuroepithelial cells, NECs) in the embryonic stage since extra cell divisions would lead to an exponential increase in the final size and thus a few regulatory changes governing the cell cycle could drive cerebral expansion [98, 99]. Such a process has been phrased as “a small step for the cell, a giant leap for mankind” [98]. A few case studies have shown consistent results, reporting that regulatory evolution contributed to a longer and faster proliferation phase of human NECs [100–104]. A modified version of the radial unit hypothesis emphasizes the increase in a derived stem cell population called outer or basal radial glial cells (oRGs or bRGs) in the fetal stage [105–109], when an excess of PSGs are expressed (Fig. 6). Interestingly, our analyses reassert the importance of the embryonic stage but showed a picture different from a few evolutionary changes proposed in the original hypothesis: the evolution of hundreds of genes appears to occur where multiple aspects of the cell cycle (e.g., DNA replication/repair) are involved and coding changes or the origination of new genes occurs in addition to regulatory changes (Fig. 6). These results expanded upon the previous case studies where positive selection acts on genes related to DNA repair or spindle functions [*BRCA1* or *ASPM*, [110, 111]]. System-level rewiring occurs in the embryonic stage because the brain develops with the highest proliferation rate [112–114]. Dramatic proliferation induces cell cycle dysfunctions such as DNA damage and replication stress in the brain [111, 115] and the majority of congenital abnormalities occur in this stage [85]. Thus, a specialized cell cycle program gets molded under positive selection where *DDX11*, *BRCA1*, or *ASPM* could support accelerated proliferation more efficiently than their ancestral forms did. In parallel, tumors also face cell cycle dysfunction due to their similarly proliferative nature [78, 116], which necessitates the co-option of cell cycle-related genes expressed in the embryonic cerebrum.

Notably, although the atavism and antagonistic pleiotropy hypotheses jointly predict that cell cycle-related PSGs derived from UC genes are necessary to be hitchhiked by tumors, whether hitchhiking occurs depends on chance. That is, different tumors have different landscapes of genetic variation and epigenetic plasticity, which causes variable chances for PSGs to be upregulated. For example, PSGs seem to be strongly upregulated in KIRC and downregulated in THCA (Fig. 2a, b). Both SCNA and promoter hypomethylation rarely occur in THCA [117, 118], possibly resulting in the lack of global upregulation of PSGs. In contrast, since KIRC is subjected to a low level of SCNA and a moderate level of hypomethylation [117–119], the strong upregulation of PSGs could also be contributed by other layers of regulation (e.g., histone modification).

## Conclusions

Overall, as atavism and antagonistic pleiotropy jointly predict, PSGs, especially those UC genes derived cell cycle-related PSGs, contribute to the embryonic cerebral development at the cost of elevated tumor risk. Thus, our meta-analyses hint on both the upsides and downsides of human evolutionary heritage.

## Methods

### Compilation of a unified gene age dataset

The gene age dataset was extracted from the synteny-based GenTree database [based on Ensembl v73, [40]] and further complemented with phylostratigraphy data generated in [23]. Although multiple phylostratigraphy datasets were generated in human [19–23], we chose [23] considering the relatively recent release time and data availability, and so as to reproduce the patterns (upregulation of UC genes and downregulation of EM genes) in [23].

As introduced in the Results, GenTree takes advantage of genome-wide synteny and performs gene-level dating parsimoniously along the vertebrate phylogenetic tree, while phylostratigraphy performs family-level dating and can be used to infer the age of ancient genes when synteny become degenerated [40]. We excluded genes for which GenTree and phylostratigraphy produced conflicting age data. Genes from the phylostratigraphy dataset that could not be mapped to Ensembl v73 due to database version differences and genes that lacked age information in GenTree due to difficulty in inferring synteny [e.g., genes situated in transposon-rich regions or assembly gaps in the outgroup species, [40]] were also discarded. We further divided the remaining genes postdating the vertebrate split in GenTree into 6 clades with at least 200 genes for each group (age group 9–14, Fig. 1b). For genes assigned to vertebrate common ancestors (the oldest age group in GenTree), we divided them into group 1–8 based on the phylostratigraphy analysis (Additional file 1: Fig. S1a). In addition, for synteny-defined PSGs, we also analyzed their age distribution in the phylostratigraphy dataset [23] in order to test whether PSGs potentially involved in the cell cycle represent duplicates derived from gene families with UC genes as the founding members.

Note that our synteny-based dating strategy is conservative in terms of identification of young genes. That is, we previously performed extensive manual curation and found that we tend to assign genes to older ages and generated smaller young gene dataset compared to other synteny-based work in humans or flies [40, 53].

For each PSG, we downloaded our previously generated origination mechanism information [40]. In brief, we classified them as duplicate (with a paralog available) and potential *de novo* genes (without a paralog). The parental copy is defined as the most similar paralog with an age older than the focal PSG. The *K*_a_/*K*_s_ values were also from the previous work [40].

### TCGA multi-omics data retrieval

We downloaded multiple datasets generated by the TCGA project with standard pipelines [46]. Specifically, we retrieved paired-end RNA-seq data from the Genomic Data Commons (GDC) data portal [120] to increase the chance of unique mapping and thus the accuracy of paralog quantification. We focused on primary tumor sample from 13 solid cancer types [marked by TCGA barcodes “01”, https://docs.gdc.cancer.gov/Encyclopedia/pages/TCGA_Barcode] and required tumor sample number not lower than 150 and normal sample number not lower than 15. We downloaded DNA methylation and SCNA data from the Broad Firehose data browser (https://gdac.broadinstitute.org/). We retrieved progression-free interval (PFI) information from [121], which was presumably more updated than those originally generated by TCGA. We finally estimated tumor purity based on expression data by following [122] and set 0.7 as the cutoff for high purity tumor samples [123].

### TCGA and normal adult tissue RNA-seq transcriptome data analysis

We quantified gene expression via the mapping-free software kallisto version (v) 0.43.1 which shows rapid speeds and decent performance on highly similar paralogs [97, 124, 125]. Note that almost all (>99%) TCGA RNA-seq samples were from different patients with one sample from one individual. For the remaining cases, more than one sample could be from one patient and we randomly selected one sample for downstream analyses to keep consistency with other samples. Since our analyses depended on Ensembl v73 [126] based gene age data compiled in the GenTree database [40], we herein used GENCODE gene annotation v18, which corresponds to Ensembl v73. We summed the estimated counts and transcripts per million mapped (TPM) values across transcripts of the same gene.

To estimate the expressional change of PSGs in tumors relative to normal samples, we performed two complementary analyses. First, we implemented the widely used limma package v3.34.9 to calculate the log_2_-based fold change (log_2_FC) in each cancer type after performing routinely used upper-quantile normalization and voom transformation [127]. Second, we carried out single sample gene set enrichment analysis (ssGSEA) with the commonly used gsva package v.1.26.0 in R [61, 62]. In this framework, the TPM values were rank normalized in each sample, and the enrichment scores were calculated by summarizing the difference in rank distribution within the gene set of interest. For Fig. 2b and c, we rescaled the raw enrichment scores within each age group as follows: (1) divide each raw score by the range between the maximum and minimum scores to make the distribution similar across age groups; (2) calculate medians for tumor and normal samples; and (3) subtract the median value of normal samples. We repeated the analyses with only the high purity samples (Additional file 1: Fig. S2a-c).

Note that we used upregulation to enrich PSGs more likely involved in tumors since genes beneficial for tumors (e.g., UC genes) tend to be upregulated in tumors. In contrast, other PSGs especially those downregulated PSGs may repress tumors as did by genes emerging in early metazoan evolution (EM genes).

In addition, we downloaded RNA-seq data from Human Protein Atlas (HPA), which spanned 26 adult tissues with at least three replicates for each tissue. Based on HPA transcriptome data across 26 tissues, we adopted the τ index to measure tissue bias of gene expression profiles [128] and took 0.8 as the cutoff to define tissue-biased genes, since 0.8 represents a border value in the distribution of τ values. Tissue-biased genes were classified according to their corresponding top tissue and Additional file 1: Fig. S1b was plotted accordingly.

### Filtering protein-coding gene models

To further increase the quality of the GENCODE v18 or Ensembl v73 coding gene annotation, we implemented multiple filters: (1) we retained gene models with the biotype tag “protein coding” and excluded transcripts shorter than 150 bp; (2) we removed genes sharing a sequence identity higher than 97.5% with the closely related paralog, since such a cutoff ensures an average of two or more unique nucleotides for each gene in TCGA paired-end RNA-seq data (length of ~50 bp); (3) based on HPA data, we removed genes with low expression [TPM < 0.2 [129]] across 26 adult tissues; and (4) we further discarded unexpressed genes [those with no reads mapped in more than 20% of samples within a cancer type [130]] across all 13 cancer types. With these filters, 18195 genes were retained for subsequent analyses. Note that majority (16,130 or 88.7%) of these genes were covered by our unified gene age dataset.

### SCNA and methylation analyses

We downloaded gene-level SCNA data identified by the GISTIC2 package [131].

To quantify the methylation level, we processed the value of each probe. Specifically, we excluded probes with more than 5% of samples harboring missing data (NA value) within a cancer type due to technical issues (e.g., multiple mapping probes). We focused on promoter methylation and analyzed probes that mapped to the 2000 bp assumed promoter region (1500 bp upstream and 500 bp downstream of the transcription start site, TSS) [132]. Herein, we defined the TSS of each gene based on the functionally most important transcripts or principal transcripts annotated by the APPRIS database [133]. In the absence of a principal transcript, we used the longest transcript. For a gene with multiple probes, we retained only the one with the strongest negative correlation between the methylation level and gene expression [134].

We implemented the Spearman correlation-based analysis to evaluate how strongly gene expression is affected by SCNA or promoter methylation. For each gene, we calculated the correlation between gene expression and SCNA/methylation within each cancer type. According to the genome-wide distribution of the correlation coefficient, 0.3/-0.3 was selected as the cutoff to define significantly correlated genes for SCNA/promoter methylation (Fig. 3). We also tested a more stringent threshold of 0.4/-0.4 and largely reproduced the patterns generated with the 0.3/-0.3 cutoff (Additional file 1: Fig. S3c).

In addition, we examined whether genes upregulated in tumor compared to normal samples show lower normal expression compared to those downregulated in tumor. We recorded the mean expression values of normal samples within each cancer type and then pooled all data across the 13 cancer types.

### Functional analyses of upregulated PSGs

We first identified pan-cancer upregulated genes. Specifically, within each cancer type, we used the limma package v3.34.9 [127] to identify genes that were differentially expressed between tumor and normal samples with a false discovery rate (FDR) lower than 0.05. We further filtered this list to select genes with absolute log_2_FC or |log_2_FC| ≥ 0.4. To define pan-cancer upregulated genes, we required that genes were upregulated in at least three cancer types and the number of cancers with upregulation was 3 times higher than that of downregulated cancer types. Pan-cancer downregulated genes were defined analogously. We also tested a more stringent |log_2_FC| cutoff of 0.6 and the patterns shown in Fig. 2e were largely unchanged as in Fig. S2d.

Since correlation-based analyses including survival and coexpression analyses depend on expressional variance, they are not appropriate for genes unexpressed in too many samples within a cancer type. Therefore, we followed [130] and excluded genes without any reads mapped across at least 20% of samples in a cancer type of interest.

We implemented the Cox proportional hazards model with the survival package v2.43.3 in R to define prognostic genes as those whose expression levels were significantly associated with the survival time of patients after controlling for clinical variables (age/stage/gender) if applicable (Additional file 2: Table S3). Among multiple clinical endpoints, overall survival (OS) and PFI have been recommended for high-quality survival analyses [121]. Note that PFI data is recommended for all 13 cancer types covered in this study, and OS data is problematic for three cancer types, where the endpoint needs a longer follow-up or the number of events is not sufficient. We thus used the PFI in subsequent analyses. We defined prognostic genes as those whose expression was significantly correlated with PFI (FDR < 0.05). The favorable or unfavorable prognostic genes were classified according to the sign of the hazard ratio, where a positive ratio indicated that the gene was associated with an increased risk of tumor progression, or vice versa. To define the overall trend of each gene, we subtracted the number of cancer types with favorable signals from the number of those with unfavorable signals. Genes were then divided into four subgroups: unfavorable (positive net number), favorable (negative), intermediate (with net value being zero), and non-prognostic (lacking prognostic signal across 13 cancer types). Since it is known that the number of prognostic genes identified by Cox analyses showed magnitude-level differences across tumor types [135], we also followed [136] and extracted the top 1500 genes most correlated with the PFI as prognostic genes within each cancer type. The pattern (Fig. 4a) remained robust (Additional file 1: Fig. S4b).

Notably, in Additional file 3 and Additional file 1: Fig. S4a/c, we also showed the hazard ratio together with the 95% confidence interval. The hazard ratio is similar to the odds ratio and both of these values are often perceived as relative risk [137]. The major difference is that the hazard ratio is not constant while the odds ratio is. Therefore, the hazard ratio is more suitable for survival data analyses [137].

To infer the functions of PSGs, we performed expression correlation-based enrichment analyses. For a given gene, we used the “ppcor” function in the R package psych v1.8.12 to calculate the Spearman correlation coefficient between its expression and that of other protein-coding genes while controlling for tumor purity within each cancer type. We took the median Spearman correlation coefficients across cancer types as a summary value for each gene pair (query and partner). To define the correlated partners, we retained genes with an absolute correlation coefficient above 0.4. With more stringent cutoffs such as 0.5 or beyond, the number of coexpressed partner genes decreases dramatically (from a median of 249 to 24 or even lower) and makes the subsequent enrichment analyses unfeasible. We further removed those partners that were located on the same chromosome as the given gene since genes located in chromosomal proximity may tend to be coexpressed regardless of their functions. We then downloaded 50 hallmark (biological processes) gene sets curated by MSigDB [67]. For each gene, we calculated the enrichment of its correlated genes across 50 hallmarks. We defined highly enriched hallmarks as those with statistical significance (odds ratio > 1, binomial test *P* < 0.05). With these steps, we assigned each query gene to zero or multiple significant hallmarks and tested whether the proportion of uPSGs assigned to one hallmark differed from that of the genomic background. For uUC and dEM genes, we performed a similar enrichment analysis (Additional file 1: Fig. S4d). Since *E2Fs* are master regulators of the cell cycle and may regulate thousands of target genes [78, 81], we removed genes potentially regulated by E2Fs, i.e., those with E2F binding sites in the promoter region annotated by MSigDB. A similar pattern was reproduced (Additional file 1: Fig. S4e).

In addition to MSigDB, we analogously mapped partner genes to DAVID functional terms [69] and reproduced similar patterns. Specifically, although DAVID is not a cancer-oriented annotation system like MSigDB, it could perform enrichment analysis across multiple ontologies simultaneously and cluster the results. Since DAVID supports numerous ontologies, we chose all five default biological process or pathway-oriented ontologies including “UP_KW_BIOLOGICAL_PROCESS,” “GOTERM_BP_DIRECT,” “BBID,” “BIOCARTA,” and “KEGG_PATHWAY.” In complement, we chose another two popular ontologies including “UP_KW_MOLECULAR_FUNCTION” and “GOTERM_MF_DIRECT.” Clusters with enrichment scores not lower than 3 were considered and the most significant term within the cluster was retained. Different from MSigDB, which has three specific terms related to the cell cycle (mitotic spindle, G2/M checkpoint, and E2F targets), we need to extract cell cycle-related terms across seven ontologies in DAVID. To simplify this process, we searched the G2/M checkpoint gene set of MSigDB in DAVID and identified 21 significant cell cycle-related terms (e.g., KW−0131~Cell cycle or GO:0005819~spindle, Additional file 2: Table S5). We also tested mitotic spindle or E2F target gene sets of MSigDB and retrieved a smaller subset of terms, which are largely covered by the previous 21 terms. We thus examined whether the coexpressed partner genes of a query gene were enriched in the 21 terms. Different from MSigDB-based analyses, which were performed locally, we had to access the web server of DAVID to enable the enrichment analyses by uploading a list of partner genes for each individual query gene. Considering the limitation of website visit, we thus selected one positive control gene set (MSigDB mitotic spindle, G2/M checkpoint, and E2F targets) including 552 genes, and one negative control gene set including 804 genes. To represent the general scenario, the later list includes eight randomly sampled MSigDB gene sets, which covers all major categories of biological processes except cell proliferation and DNA damage (these two categories being related with cell cycle, Additional file 1: Fig. S4d): metabolic (bile acid metabolism), cellular component (apical junction), immune (complement, coagulation), pathway (protein secretion), signaling (androgen response, mTORC1 signaling) and development (epithelial-mesenchymal transition). We then used the RDAVIDWebService package v1.28.0 [138] to enable enrichment analyses for 1356 (552+804) times.

To validate the excess of cell cycle-related PSGs based on MSigDB or DAVID, we further analyzed an independent cell cycle gene list, which is curated via a literature survey and experimental analyses in normal cells [70]. This list includes 701 genes involved in a dozen distinct processes, such as cell cycle progression, DNA damage/replication, or spindle assembly [70]. Despite of database version changes, the majority (666 or 95.0%) of these genes were covered in our analyses.

For CRISPR/Cas9 screening data analyses, we downloaded the single guide RNA (sgRNA) abundance fold changes file (2019_Q4 version) from the DepMap data portal [71]. To determine the correspondence between TCGA cancer types and DepMap cell lines, we downloaded the cell line annotation file from the Cancer Cell Line Encyclopedia (CCLE) project [139] and retained cell lines that corresponded to the 13 cancer types covered in this study. Considering within-tumor heterogeneity [140], we required at least five cell lines to represent a cancer type. Thus, 138 cell lines covering 9 cancer types were considered in the downstream analyses. To control for the multiple mapping of sgRNAs, we reprocessed the screening data. We mapped the sgRNA sequences to human reference genome (GRCh38) with bowtie v1.2.2 [141] and retained 16424 genes with unique sgRNAs mapped without any mismatch. Here, “uniqueness” was defined as at least two mismatches to other protein-coding genes and at least one mismatch to noncoding regions. To normalize the sgRNA fold changes across cell lines, we scaled the median fold changes of predefined essential genes as -1 and the median fold changes of predefined nonessential genes as 0 [142]. We calculated the gene-level dependency scores as the medians of sgRNA abundance fold changes between replicates. Since DepMap identified a median of 11.7% genes as essential genes across hundreds of cell lines (Additional file 1: Fig. S4h), we chose a fold change cutoff in each cell line to extract the top 11.7% of genes as essential. To define common dependencies, we followed the methodology described in [72]. In brief, for each gene, we calculated its rank in terms of scores within each cell line and recorded the rank value corresponding to the 90th percentile. In other words, we recorded the relative lowest rank for each gene in terms of its dependency. We then plotted the density distribution of the transformed rank values across genes (Fig. 4f). Since 6.2% of genes were classified as common dependencies in [72], we chose a cutoff to extract the top 6.2% (1018) of genes.

### Knockdown experiments in cancer cell lines

We cultured two commonly used cell lines and transfected siRNAs targeting *DDX11*. Specifically, we purchased A549 and HCT116 cell lines from the American Type Culture Collection (ATCC) and confirmed their identity with short tandem repeat (STR) analysis. We carried out mycoplasma testing with One-step Quickclolor Mycoplasma Detection Kit (Shanghai Yise Medical Technology Co., Ltd.) to rule out mycoplasma contamination. A549 and HCT116 cells were then cultured in RPMI-1640 medium with 10% fetal bovine serum (Thermo Fisher Scientific) at 37 °C and 5% CO_2_. We purchased the siRNA targeting *DDX11* and the scrambled oligonucleotide (negative control siRNA) from GenePharma by following the siRNA sequences reported in [143], i.e., CCTGTGTCTGTCTTCTTCCTGCGAA. Note that this published siRNA sequence is perfectly mapped to *DDX11* while harboring one mismatch against *LOC642846* and *DDX12P* and at least three mismatches against other locations in the genome. The control siRNA sequence is “CAGTCGCGTTTGCGACTGGC,” which cannot be mapped to any site in the human genome. We seeded 2 × 10^5^ cells or 3 × 10^3^ cells into each well of 6-well (for qRT-PCR) or 96-well (for cell proliferation assay) plates, respectively. We conducted siRNA transfection with Lipofectamine RNAi MAX (Thermo Fisher Scientific) one day after cell seeding with the final concentration of 20 nM. We carried out three independent transfection experiments.

We then quantified the knockdown efficiency with qRT-PCR. We first extracted total RNA with RNeasy Plus Mini Kit (Qiagen) 2 days after siRNA transfection. For each sample, we reverse-transcribed 1 μg of total RNA into cDNA with the High-Capacity cDNA Reverse Transcription Kit (Thermo Fisher Scientific) by following the manufacturer’s protocol. We performed qRT-PCR experiments with PowerUp SYBR Green Master Mix (Thermo Fisher Scientific) in triplicate in 20 μl reactions on a Stratagene Mx3005P system (Agilent Technologies). The primers synthesized by Biomed were as follows (5′-3′): *HPRT1* (a housekeeping gene used as the internal control as in [143]) (forward: TGACACTGGCAAAACAATGCA; reverse: GGTCCTTTTCACCAGCAAGCT) and *DDX11* (forward: CACAACCTGATCGACACCAT; reverse: CTTCCCGTATCGCTCCAC). Note that the primers mapped perfectly to *DDX11* but harbored one to two mismatches for *LOC642846* and *DDX12P*. We calculated the relative expression via the 2^–ΔΔCt^ method and normalized the expression levels against that of *HPRT1* [143].

To measure the consequence of *DDX11* knockdown, we performed Incucyte S3 (Essen BioScience) live-cell analysis as follows: (1) for each siRNA, we performed replications with six wells; (2) after transfection, cell proliferation was monitored by analyzing the occupied area (% confluence) of cell images over time; and (3) during cell proliferation, the graphs from the phase of cell confluence area were recorded over a 6-hour interval for 72 hours after transfection according to the manufacturer’s instructions.

### Evolutionary analysis of *DDX11*

To dissect the evolutionary history of *DDX11*, we examined the syntenic alignment at both loci (*LOC642846/DDX12P*, *DDX11*) provided by the UCSC genome browser [144]. We chose five primates, namely, humans, chimpanzees, gorillas, orangutans, and rhesus monkeys, considering both phylogenetic relationships and the quality of genome assemblies. Given the presence and absence of orthologous loci across these species, we reconstructed the evolutionary history of this gene family by following the parsimony rule.

To infer the selection force acting on *DDX11* homologs, we extracted the homologous sequences of 10 phylogenetically representative mammals from the Ensembl database [126]. *LOC642846*/*DDX12P* in humans and *DDX12P* in chimpanzees were conceptually translated with the DDX11 protein of humans as the template. We implemented PRANK v170427 to perform protein-level alignment since this software generates fewer alignment errors [145]. Protein alignment was transformed to codon level alignment with PAL2NAL v14 [146]. As described in previous studies [79, 147], the *DDX11* protein sequence was divided into five functional regions (T, Arch, Fe-S, Hel, and Linker), in which Hel denotes all known small motifs. We extracted the alignment for each region and then conducted a branch test with the codeml program in the PAML v4.9h package to detect the signal of natural selection [84]. For each domain, the significance test was performed by comparing two-ratio and three-ratio branch models, where ω_0_, ω_1_, and ω_2_ denote the rates (*K*_a_*/K*_s_) of the outgroups, pseudogenic copies and derived functional copies, respectively (Additional file 1: Fig. S6e). Then, we ran codeml based on the following hypotheses: H1, (ω_0_ = ω_2_) ≠ ω_1_; H2, (ω_0_ = ω_1_) ≠ ω_2_; and H3, ω_0_ ≠ ω_1_ ≠ ω_2_. A likelihood ratio test was performed between H1 and H3 or H2 and H3 to evaluate the statistical significance of differences between the outgroup and the newly derived copies/pseudogenic copies, respectively.

We also analyzed how *DDX11* evolved at the expression level. To this end, we took advantage of a previous study, which profiled four major organs (brain, testis, liver, and heart) across four phylogenetically representative mammals (human, chimpanzee, rhesus monkey, and mouse) with high-depth strand-specific RNA-seq data [148]. We removed low-quality reads with Trimmomatic v0.39 [149], mapped the remaining reads to the reference genome with STAR v2.6.1d [150], and performed the quantification with RSEM v1.3.1 [151]. Fragments per kilobase million (FPKM) values were log_2_ transformed after adding one. We used the STAR/RSEM pipeline here rather than kallisto because this dataset is small and the STAR/RSEM pipeline has a slightly better performance despite slower running speed [97].

To account for between-species heterogeneity in gene annotations and sequencing libraries, we implemented the following procedures. On the one hand, since both the N-terminal and C-terminal domains of DDX11 protein are required for its function [76, 152], we used only annotated protein-coding transcripts with protein products of at least 800 amino acids across species (Additional file 2: Table S6). On the other hand, we downloaded orthologous information from Ensembl BioMart v98 [153] and retained one-to-one orthologous genes across species. After removing genes showing tissue-biased expression in humans (Fig. 1b), we used the median expression level of the remaining 9308 presumably housekeeping genes as the internal control. For each tissue and each species, *DDX11* expression was normalized by dividing the corresponding median value (Fig. 5f).

### Developmental transcriptome data analyses

To examine the expression dynamics of the developing brain, we took advantage of a strand-specific RNA-seq dataset [90]. We reprocessed the raw sequencing data by following the aforementioned TCGA gene quantification pipeline except that the “strand-specific” mode in kallisto v0.43.1 was enabled. We divided 52 samples of cerebrum (forebrain) into 12 stages of development according to [113] with the requirement of at least two replicates for each stage (Table S7). Medians of gene expression values within each stage were subsequently used. We removed weakly expressed genes (median expression level lower than 0.2 across all 12 stages). Here, weeks after conception conventionally refers to completed weeks [154]; thus, the end of the embryonic stage, i.e., 7 postconceptional weeks (PCWs), corresponds to 56 days postconception [85]. To determine the stage at which a given gene is preferentially upregulated, we transformed the expression values as *Z*-scores by subtracting the mean expression level and dividing by the standard deviation. For each gene, we defined the upregulated stage according to the following two criteria: (1) the gene of interest showed the highest expression level in this stage; and (2) the *Z*-score > 1.2 (1.2 representing the 90% quantile of *Z*-score distribution, Additional file 1: Fig. S7a). We used the other six organs including the cerebellum (hindbrain), heart, kidney, liver, ovary, and testis as the controls. Note that these somatic or reproductive tissues represent all three germ layers and thus serve as a comprehensive control to establish relevance to uniquely enhanced cell cycle activity in embryonic cerebral (forebrain) development.

### Meta-analysis of genes with biased expression in the embryonic brain

For 4553 genes upregulated in the embryonic stage, we performed enrichment analysis with Metascape v3.5. Since the size limit of the input gene list was 3000, we thus randomly sampled 3000 genes 5 times and retained four GO terms that occurred at least 4 times, ranking in the top 5 in each sampling process (Additional file 2: Table S8). This is also why we chose the top 4 instead of the often used top 5 terms.

We downloaded the latest list of human protein-coding genes with signal of positive selection in the coding region of human lineage, which was based on the modified *K*_a_/*K*_s_ test after correcting for GC content in primates [155]. Genes with promoter regions subjected to positive selection in human lineage were also compiled from [156] and only genes with *P*-values less than 0.05 were selected. Notably, numerous lists of noncoding regions under positive selection or human/primate-specific regulatory sequences have been published [108, 157–161]. However, these lists were derived from functional genomics data of fetal or postnatal brains and thus could not be directly transferred to the embryonic brain. This is why we ultimately used the list generated in [156], which was computed based on the human genome directly and thus not anchored to a specific developmental stage.

For all these meta-analyses, we used the Ensembl ID or gene symbol to cross-reference different datasets.

### Figures and statistical tests

We used violin plots to show the data distributions. The bar indicates the interquartile range (IQR) and the point indicates the median.

We estimated significance with Wilcoxon signed-rank tests, *t*-tests, chi-square tests, and binomial tests depending on the specific contexts. Unless otherwise specified, we implemented two-sided tests. Multiple test correction was performed via the FDR strategy as implemented in the p.adjust function in R v3.4.4. All relevant R-based packages are summarized in Additional file 2: Table S10.

## Supplementary Information

Additional file 1: Figure S1. Characterization of primate-specific genes (PSGs). Figure S2. Pan-cancer upregulation of PSGs. Figure S3. Factors underlying up- or downregulation of genes in tumors. Figure S4. Survival, hallmark enrichment and cell line screening data analyses. Figure S5. Functional characterization of *DDX11* in cancer samples or cell lines. Figure S6. Evolution of *DDX11*. Figure S7. Additional analyses of genes expressed during the development.

Additional file 2: Table S1. Gene age and expression data across 13 tumor types. Table S2. ssGSEA values across tumor and normal samples in 13 cancer types. Table S3. Clinical covariant in the survival analysis. Table S4. Annotation of upregulated PSGs (uPSGs). Table S5. 21 Terms used in DAVID analysis. Table S6. Full-length protein-coding transcripts of *DDX11* in 4 species. Table S7. Cerebral RNA-seq transcriptome. Table S8. GO enrichment analysis results. Table S9. Positively selected genes used in this project. Table S10. R packages used in the project.

Additional file 3. Results of survival analysis within 13 cancer types.

Additional file 4. Peer review history.

## Data Availability

No large-scale experimental data has been generated in this project. The age data used in this article were downloaded from the previous literatures. In brief, the synteny-based gene age data was from GenTree database [162] and the phylostratigraphy data was from Supporting Information (Dataset S1) of one previous work [163]. Additional file 3 and the R code for Figs. 1, 2, 3, 4, 5, and 6 are available at both GitHub [164] and Zenodo [165].
