# Supplementary material for: Pan-cancer surveys indicate cell cycle-related roles of primate-specific genes in tumors and embryonic cerebrum
Source: Genome Biol. 2022 Dec 6;23:251. doi: 10.1186/s13059-022-02821-9 (PMC9724437; doi:10.1186/s13059-022-02821-9)
Supplement: Supplementary file 1 — Additional file 1: Figure S1. Characterization of primate-specific genes (PSGs). Figure S2. Pan-cancer upregulation of PSGs. Figure S3. Factors underlying up- or downregulation of genes in tumors. Figure S4. Survival, hallmark enrichment and cell line screening data analyses. Figure S5. Functional characterization of DDX11 in cancer samples or cell lines. Figure S6. Evolution of DDX11. Figure S7. Additional analyses of genes expressed during the development. [file 13059_2022_2821_MOESM1_ESM.pdf]

Supplementary Figures

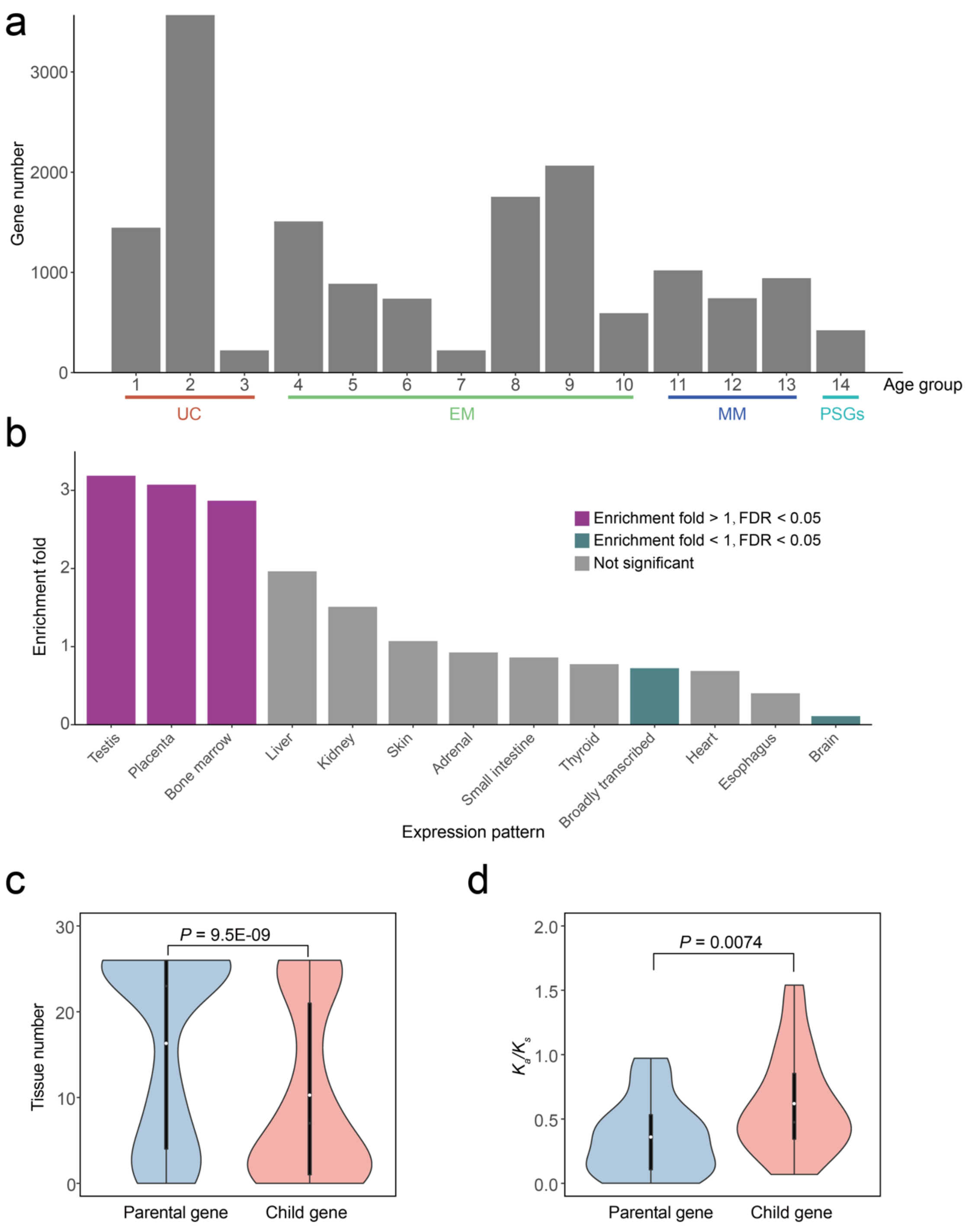

Fig. S1 Characterization of primate-specific genes (PSGs). a Distribution of gene counts in each age group. **b** Distribution of tissue-biased PSGs. For each group, the enrichment fold was defined as the ratio of its proportion in PSGs divided by the overall genomic proportion (see also Methods). A binomial test was implemented, and only groups with more than 100 genes were included in this

analysis. PSGs are overrepresented in testis/placenta/bone marrow biased genes and underrepresented in broadly transcribed genes or adult brain-biased genes. Both patterns are consistent with previous reports [35,40,97]. **c** Comparison of expression breadth between PSGs and their parental copies. Tissue number denotes the number of tissues in which the expression level (TPM) is higher than 1. **d** Comparison of evolution rate (K_a/K_s) between PSGs and the parental copies. For Panels c and d, one-sided Wilcoxon signed-rank test was implemented.

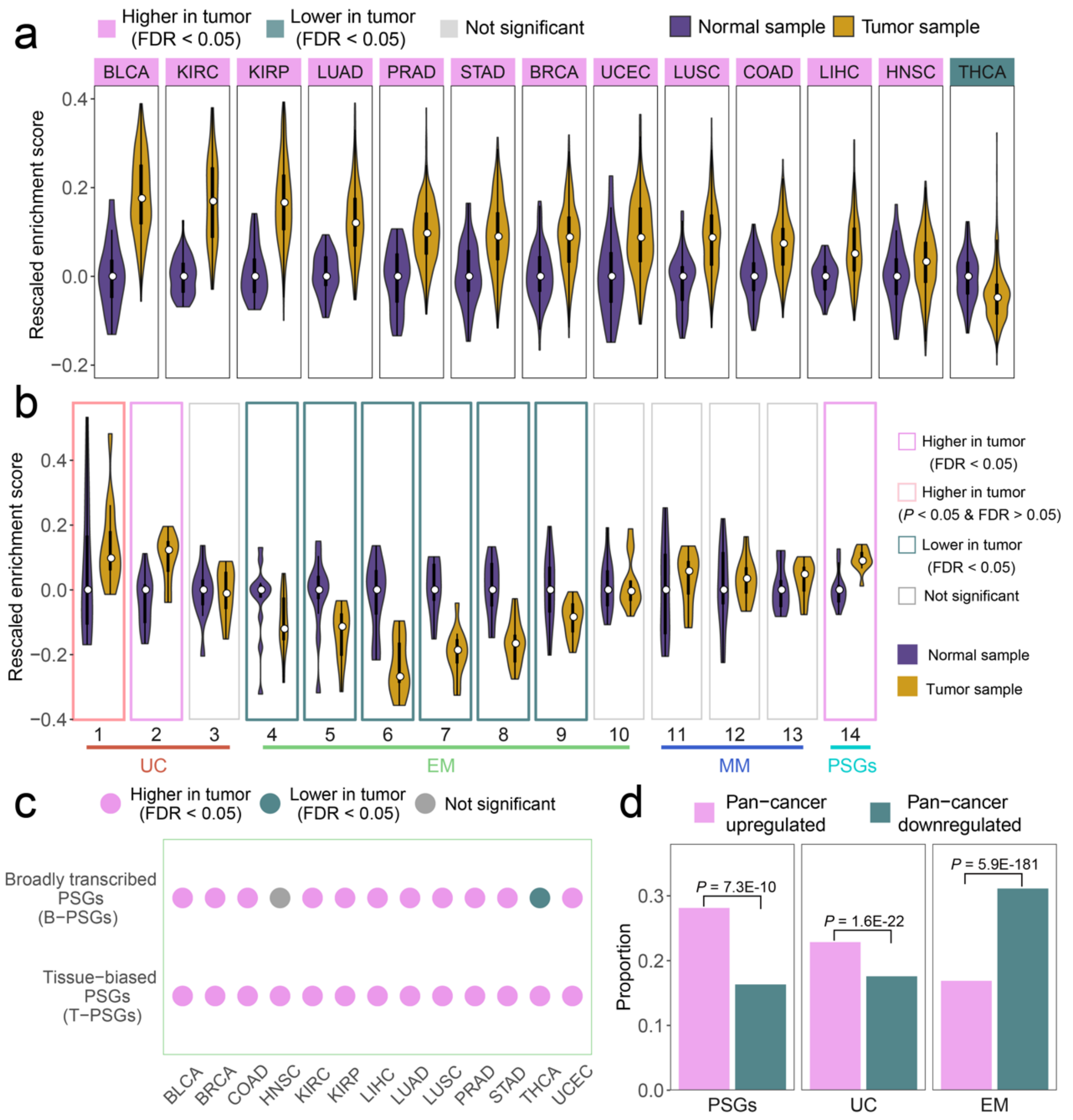

Fig. S2 Pan-cancer upregulation of PSGs. a-c Distribution of enrichment scores: PSGs (**a**), 14 age groups (**b**), B-PSGs and T-PSGs (**c**). Only high-purity tumor samples are used in these three panels with figure conventions following Fig. 2b-d, respectively. **d** Proportion comparison between pan-cancer upregulated and downregulated genes. This panel follows the same convention as Fig. 2e except that a more stringent cutoff was used to define the differentially expressed genes (Methods).

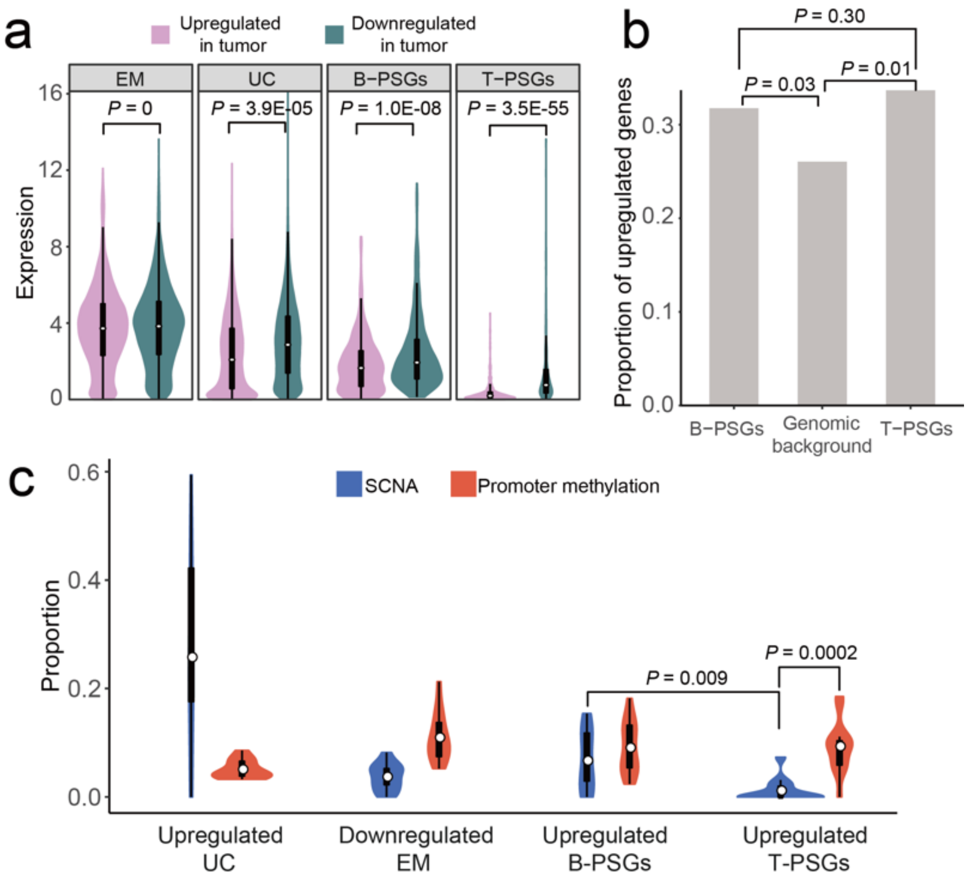

Fig. S3 Factors underlying up- or downregulation of genes in tumors. a Comparison of normal expression levels between genes up- and downregulated in tumors. **b** Comparison of pan-cancer upregulated gene proportion. **c** Pan-cancer proportion distribution of genes whose expression is significantly correlated with SCNA or promoter methylation. This panel follows the same convention as Fig. 3 except that a more stringent cutoff of 0.4/-0.4 was used (Methods).

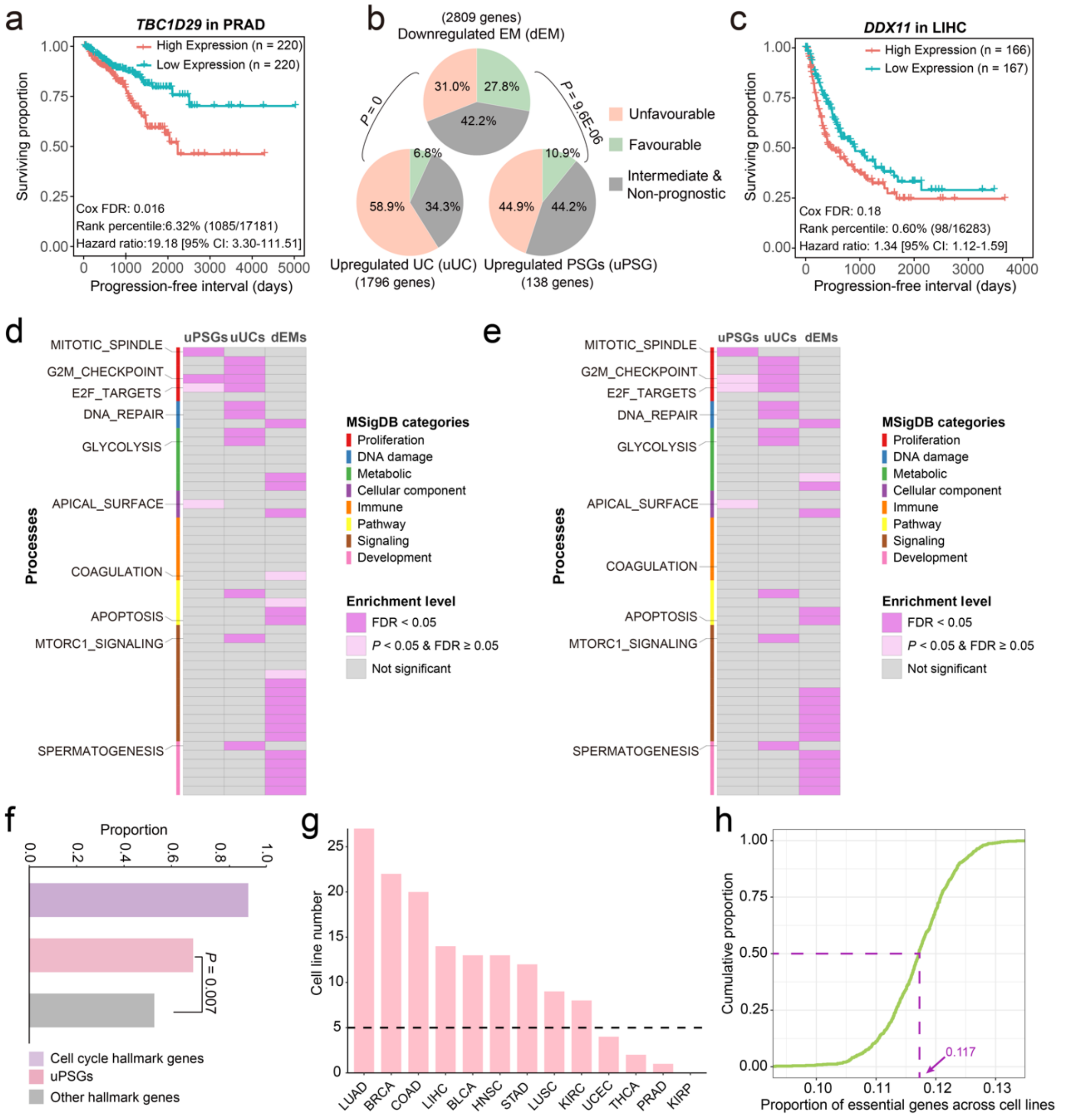

Fig. S4 Survival, hallmark enrichment and cell line screening data analyses. a The survival plot of *TBC1D29* in prostate adenocarcinoma (PRAD). **b** Stronger expression of uPSGs or uUC genes more often leads to unfavorable survival compared to that of dEM genes. This panel reproduces Fig. 4a by using the top 1500 genes most associated with the progression-free interval time. **c** The survival plot of *DDX11* in liver hepatocellular carcinoma (LIHC). **d-e** Enrichment of uPSGs, uUC genes and dEM genes in 50 annotated MSigDB hallmarks relative to the genomic background. For genes assigned to at least one MSigDB hallmark, we performed one-sided binomial test with multiple testing correction (FDR) to examine the distribution bias of these genes across hallmarks.

Hallmarks were arranged and color-coded according to a total of eight corresponding functional categories. For the proliferation hallmark category, three cell cycle related hallmarks were labeled. For the other seven categories, one representative hallmark was labeled. Panel e follows the same convention except that genes with E2F binding motifs in the promoter region were removed to avoid the interference of this key transcription factor driving cell cycle. **f** The proportion of cell cycle related genes inferred via the DAVID annotation system. Cell cycle hallmarks refer to mitotic spindle, G2/M checkpoint and E2F targets of MSigDB, while other hallmarks refer to eight randomly sampled MSigDB gene sets (*e.g.*, apical junction; Methods). These two datasets serve as the positive and negative controls, respectively. The Y-axis indicates the proportion of cell cycle related genes out of all genes assigned to at least one DAVID term. **g** Distribution of available cancer cell lines corresponding to 13 cancer types. **h** Cumulative distribution curve of the dependency (essential gene) proportion across cell lines. For each cell line, DepMap identified 10%-13% genes as essential, where the median value was 11.7% (marked as the purple arrow). We thus followed this work and extracted the top 11.7% of genes in each cell line as essential (Methods).

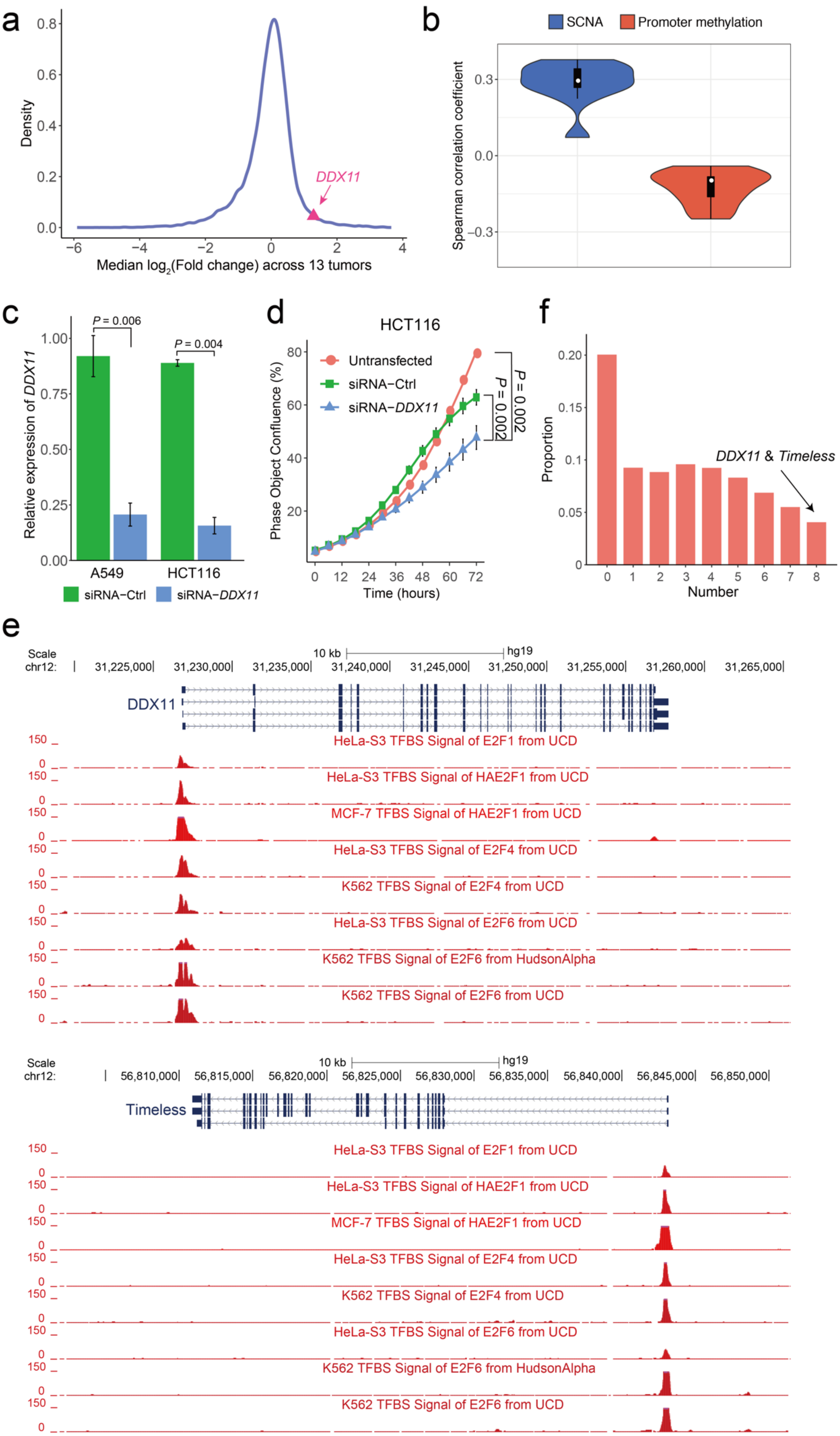
a
Density
DDX11
Median log2(Fold change) across 13 tumors
b
SCNA
Promoter methylation
Spearman correlation coefficient
c
Relative expression of DDX11
P = 0.006
P = 0.004
A549
HCT116
siRNA−Ctrl
siRNA−DDX11
d
HCT116
Untransfected
siRNA−Ctrl
siRNA−DDX11
P = 0.002
P = 0.002
Phase Object Confluence (%)
Time (hours)
f
Proportion
DDX11 & Timeless
Number
e
Scale
chr12:
10 kb
hg19
DDX11
HeLa-S3 TFBS Signal of E2F1 from UCD
HeLa-S3 TFBS Signal of HAE2F1 from UCD
MCF-7 TFBS Signal of HAE2F1 from UCD
HeLa-S3 TFBS Signal of E2F4 from UCD
K562 TFBS Signal of E2F4 from UCD
HeLa-S3 TFBS Signal of E2F6 from UCD
K562 TFBS Signal of E2F6 from HudsonAlpha
K562 TFBS Signal of E2F6 from UCD
Timeless

Fig. S5 Functional characterization of *DDX11* in cancer samples or cell lines. a Distribution of expression changes in all genes between normal and tumor samples. *DDX11* is marked. **b** Pan-cancer distribution of Spearman correlation between *DDX11* gene expression and SCNA/promoter methylation. **c** Quantification of DDX11 knockdown efficiency in terms of its expression in two cell lines. For "Ctrl" experiments, a non-targeting siRNA was used (Methods). The error bars denote the standard error of the mean (SEM) calculated based on three biological replicates. T-tests were used to quantify the significance level. **d** Time course cell count curves in HCT116 cells. The figure convention follows those of Fig. 5a. **e** The UCSC genome browser screenshot shows the ChIP-seq peaks of E2F in the promoter region of DDX11 (top) and Timeless (bottom). For the top gene model track, the thinner and thicker boxes indicate the untranslated regions (UTRs) and coding exons, respectively, while the middle lines represent introns, with zigzags showing the transcriptional orientations. For the transcription factor binding site (TFBS) signal track or ChIP-seq, the Y-axis range was set as [0, 150] to make peaks more visible. The antibody was against the HA tag for two E2F1 tracks. The similarity between individual E2F members is consistent with their somewhat overlapping function and binding preference [78,81]. **f** Genome-wide distribution of the number of E2F binding across eight samples. Fewer than 5% of genes, including DDX11 and Timeless, are constantly bound.

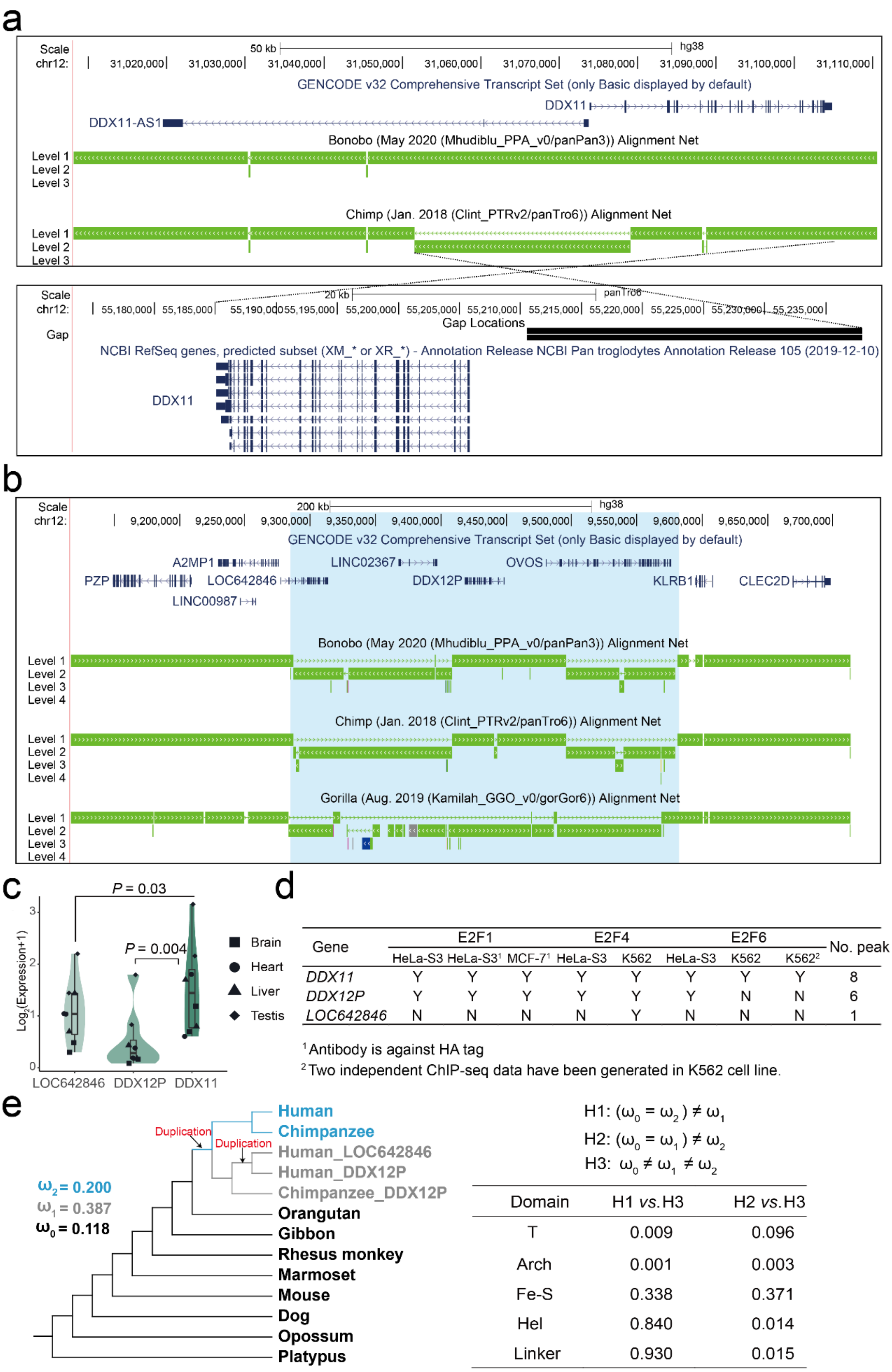

d

Gene	E2F1			E2F4		E2F6			No. peak
	HeLa-S3	HeLa-S3[1]	MCF-7[1]	HeLa-S3	K562	HeLa-S3	K562	K562[2]	
DDX11	Y	Y	Y	Y	Y	Y	Y	Y	8
DDX12P	Y	Y	Y	Y	Y	Y	N	N	6
LOC642846	N	N	N	N	Y	N	N	N	1

[1] Antibody is against HA tag
[2] Two independent ChIP-seq data have been generated in K562 cell line.

e

H1: $(\omega_0 = \omega_2) \neq \omega_1$

H2: $(\omega_0 = \omega_1) \neq \omega_2$

H3: $\omega_0 \neq \omega_1 \neq \omega_2$

Domain	H1 *vs.* H3	H2 *vs.* H3
T	0.009	0.096
Arch	0.001	0.003
Fe-S	0.338	0.371
Hel	0.840	0.014
Linker	0.930	0.015

Fig. S6 Evolution of *DDX11*. a Synteny view of the human *DDX11*/*DDX11-AS1* locus. The UCSC

genome browser uses the Net track to show the syntenic information, with the upper levels being more likely orthologous than the lower levels. In other words, the lower levels may represent one-way syntenic mapping caused by paralogs. The top panel use human (UCSC version, hg38) as the focal species, while the bottom panel uses chimpanzee (panTro6) as the focal species. Although bonobo shows a continuous synteny relative to human, the orthologous locus in its sister species (chimpanzee) harbors a sequencing gap. **b** Synteny view of the human *LOC642846*/*DDX12P* locus. The two homologs together with their flanking regions are subject to rampant rearrangements in hominoids, leading to disruption of synteny (highlighted in light blue) in bonobo, chimpanzee and gorilla. Manual curation shows that bonobo/chimpanzee only encode an inverted copy, and most sequences have been deleted in gorilla. For Panels A and B, level-2 Net just shows one-way synteny. **c** Expression profile of *DDX11* paralogs across four human tissues. **d** Summary of *E2F* ChIP-seq peaks for *DDX11*, *DDX12P* and *LOC642846*. The data were generated by the ENCODE project. Y/N indicates the presence/absence of a binding peak in the promoter region of the target gene. **e** The K_a/K_s (ω) test framework. The left panel shows the phylogenetic tree of the *DDX11* family. Black, gray and blue colors in the tree represent the outgroup, pseudogenized homologs, and newly derived group, respectively. The whole protein level K_a/K_s was estimated via a three ratio model and marked along the tree. The right table shows the Chi-square test *P* values between two models within each functional region.

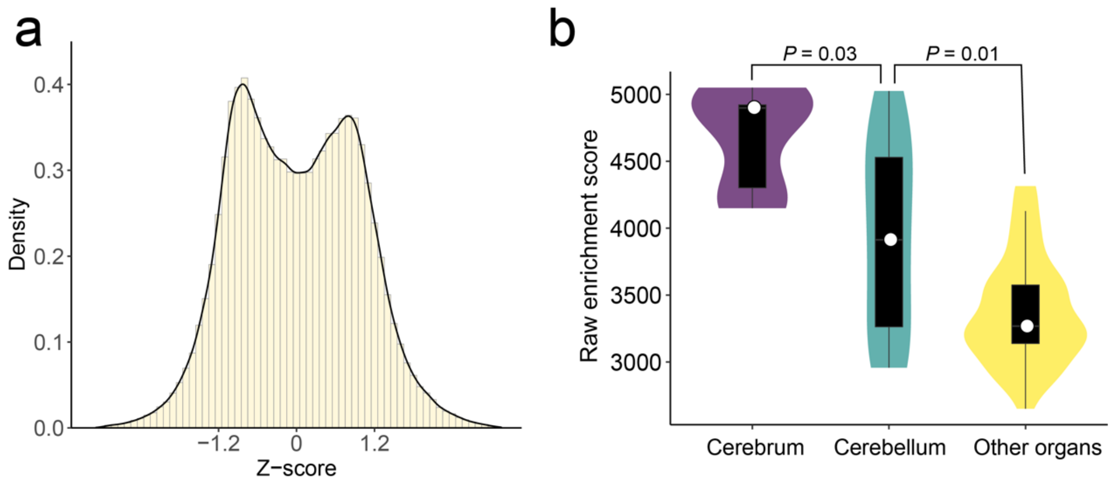

Fig. S7 Additional analyses of genes expressed during the development. a Genome-wide distribution of Z-scores. For a gene of interest, we defined a stage as the preferentially upregulated stage if this gene showed the highest expression in this stage and the Z-score was higher than 1.2 (top 10% percentile, see also Methods). **b** ssGSEA raw expression score of cell cycle related genes across different organs in the embryonic stage**.** The 666-gene list was used to define cell cycle related genes.
