# Supplementary material for: Pan-cancer surveys indicate cell cycle-related roles of primate-specific genes in tumors and embryonic cerebrum
Source: Genome Biol. 2022 Dec 6;23:251. doi: 10.1186/s13059-022-02821-9 (PMC9724437; doi:10.1186/s13059-022-02821-9)
Supplement: Supplementary file 4 — Additional file 4. Peer review history. [file 13059_2022_2821_MOESM4_ESM.docx]

**Review history**

**First round of review**

**Reviewer 1**

This paper looks at the role of primate specific genes in cancer using pan-cancer data from TCGA. While an interesting study representing a great deal of work, there are several issues with the study as presented that limit my enthusiasm for publication.
The authors never define what they mean by a primate specific gene. The gene they focus on, DDX11, has orthologs in both s. cerevisiae (32% identity and 47% similarity) and s. pombe (34% identity and 52% similarity). Ensembl Compara shows that there are 184 1:1 orthologs across 280 species, most of which have greater than 50% identity. Given this, all of the phylostratigraphy studies as well as the gene aging methodology by Cisernos, et al. would classify this gene as UC. Similar results are obtained with the 423 genes they designate as PSG. Only 58 of these genes have gene ages of less that 74 million years, the median age at which primates split from other vertebrates according to TimeTree.org. Of the remaining genes, 216 would be classified as metazoan and 94 would be classified as unicellular. Thus, while the data they present is interesting, I am not at all convinced that the main hypothesis, antagonistic pleiotropy, is supported.
There are several issues/questions surrounding some of the statistical analyses reported in the paper.
1) Many of the analyses are only reported in the figure legends, and not mentioned in the Methods section, making it difficult to determine if the correct analysis has been applied.
2) The authors need to specify both R version and package version for all R-based analyses as this is essential to ensure replicability of the analysis.
3) Any models used in the survival analysis need to be specified in the methods. While all TCGA projects were built around primary tumor samples, some of the studies, such as the bladder cancer study used in the manuscript, included multiple primary, metastatic or recurrent tumors. It is unclear whether the data was either filtered to remove these tumors or whether a variable was included to account for this. Additionally, the survival model that can be inferred from the information given does not address well-known factors that would influence PFS like tumor stage, patient age, and patient sex. The survival analyses need to be re-run taking these characteristics into account, and it would be useful to report out the OR for each variable in the model along with the 95% CI. Also, as a detail for making sure survival is understood appropriately in the paper, it should always be referred to as progression-free survival, as most readers will see survival and assume overall survival as the end point. It would also be nice to see some of the survival curves in the supplementary data. You can use Xena to generate these for some of the PSG.
4) Given that TCGA data is sample based, and samples should have gene expression data along with both copy number and methylation data, it would be more appropriate to correlate DNA copy number and/or percent methylation with gene expression for the data sets using the Spearman's correlation coefficient. This analysis will give a better idea of how much copy number and methylation contribute to expression difference than the current analysis. Additionally, the authors could do an all by all analysis across all tissues of origin and see if there are interesting relationships between genes that correspond to different age groups, for example: do you see interesting relationships between UC genes and the primate specific genes? The current analysis suggests association of amplification and hypomethylation with over-expression but does not support correlation strongly enough to justify causation. Doing the correlation analysis would demonstrate this point more forcefully. Since other factors beyond copy number and methylation status play a role in transcript abundance, reasonable correlation coefficients for self-self comparisons (e.g. gene 1 copy number and gene 1 expression) makes a much stronger argument for causation. This type of analysis also has the advantage of not needing to classify genes as amplified or deleted which gets tricky in copy number data in tumors because the cut-offs used depend on the ploidy of the tumor.
5) It is unclear which analyses were controlled for multiple comparisons. The number of analyses reported guarantees more than one result is statistically significant due to chance alone. This needs to be addressed. The p.adjust function in the stats package offers several methods for doing the adjustment for multiple comparisons.
In addition to the concerns about the details of the statistical analysis, some of the conclusions are too strong. For example, the conclusion that uPSGs "rewire" cell cycle is too strong. What can be concluded is that uPSGs play a role in proliferation and/or viability but no more because the assumption that correlated gene expression is indicative of common biological function is tenuous. To support their stated conclusions, the authors need to show that the partner genes are not potentially in a gene expression neighborhood. Gene expression neighborhoods are regions of the genome where genes have similar gene expression levels but are not involved in the same biological functions or pathways. One way to avoid including genes that are correlated by virtue of being in the same or similar gene expression neighborhoods is to be considerably stricter on the correlation coefficient filter. I would suggest that if correlated gene expression is how they want to map genes into known functions, they should use a cut-off of 0.7 or better, and then only pick partner genes that are on different chromosomes and are not regulated by E2F. E2F regulation, by virtue of being essential to cell cycle control, will result in partner genes that map most abundantly to categories related to cell cycle control. They should also use DAVID and do a functional enrichment analysis to double check the results of any functional enrichment where they map their genes to the ontology by partner gene expression. The advantage of using DAVID is that it will do an enrichment across multiple ontologies simultaneously and cluster the results. Even if the genes are not well characterized, the authors should be able to tease out the biology underlying them and correlate that to their other analyses. This type of analysis will also mitigate the confounding effect of E2F control.
In Line 374, the conclusion is too strong. E2F likely coordinates DDX11 expression in conjunction with cell cycle, but DDX1, by virtue of being a helicase involved in S-phase, may have little to do with controlling cell cycle.
Finally, the authors provide good support for these genes being enriched in brain development. The rest of the conclusions in this paper are iffy because the evidence that these genes are newly evolved is weak. The data more likely support an interpretation that selection acting on brain development has favored the creation of paralogs of unicellular genes, permitting precise control of key cellular functions in both the context of tissue and developmental stage. However, the evolution of the paralogs has not removed their responsiveness to the ancient control mechanisms, thus explaining why a reversion to unicellular biology in cancer leads to these genes being involved in tumorigenesis.

**Reviewer 2**

In this manuscript the authors explored the hypothesis of antagonistic pleiotropy by associating the gain of primate specific genes (PSGs) with cancer progression. They started with a phylostratification analysis and defined the birth time for each of the human genes. They observed generally upregulations of PGSs and UCs (unicellular ancestor genes), but downregulations of EMs (early metazoan genes), in cancers. The expression upregulations of PSGs in cancer can be ascribed mechanistically to copy number amplification or hypomethylation. Then, they showed the PSGs tend to be cell cycle related, and evolutionarily selected for human brain functions. In addition to reporting general patterns, a case gene (DDX11) was elaborated.
I found the work overall very interesting. The analyses are well done and the presentation is generally clear, though there are a few issues that should be addressed before publication.
1). The first half of the paper is generally clear, but the second half, in particular, the brain part, is confusing. The authors should focus on PSGs, instead of brain biased genes (Fig. 6).
2). The cell cycle part is rather minor, and the evidence is indirect and weak. I feel it constitutes a major obstacle for an easy read. In particular, Fig. 4 is most confusing.
3). The authors conducted a lot of analyses, some of which are not obviously necessary. In addition, too many details are described in main text. The authors shall make full use of the supplementary part and the method part to make the paper concise and fluent.
4). It's ok to state that, according to antagonistic pleiotropy, PSGs may promote cancer. But, given there are both positive and negative regulations in gene networks, why PSGs have to be upregulated to promote cancer?
5). The Fig. 2c and 2e are most interesting. Ideally, the functional characterizations of PSGs in the later parts should be accompanied by the same characterizations of the UC and EM genes.
6). Do absolute expression levels underlie the observation of up- or down-regulations. For example, lowly expressed genes cannot be down-regulated further, and highly expressed genes tend to show expression reduction.
7). Why is the grey bar wider in Fig. 6a?

**Authors’ response to reviewers**

*REVIEWER #1*

*This paper looks at the role of primate specific genes in cancer using pan-cancer data from TCGA. While an interesting study representing a great deal of work, there are several issues with the study as presented that limit my enthusiasm for publication.*

Thanks for your comments and we addressed all these issues as follows.

*The authors never define what they mean by a primate specific gene. The gene they focus on, DDX11, has orthologs in both s. cerevisiae (32% identity and 47% similarity) and s. pombe (34% identity and 52% similarity). Ensembl Compara shows that there are 184 1:1 orthologs across 280 species, most of which have greater than 50% identity. Given this, all of the phylostratigraphy studies as well as the gene aging methodology by Cisernos, et al. would classify this gene as UC. Similar results are obtained with the 423 genes they designate as PSG. Only 58 of these genes have gene ages of less that 74 million years, the median age at which primates split from other vertebrates according to TimeTree.org. Of the remaining genes, 216 would be classified as metazoan and 94 would be classified as unicellular. Thus, while the data they present is interesting, I am not at all convinced that the main hypothesis, antagonistic pleiotropy, is supported.*

*Finally, the authors provide good support for these genes being enriched in brain development. The rest of the conclusions in this paper are iffy because the evidence that these genes are newly evolved is weak. The data more likely support an interpretation that selection acting on brain development has favored the creation of paralogs of unicellular genes, permitting precise control of key cellular functions in both the context of tissue and developmental stage. However, the evolution of the paralogs has not removed their responsiveness to the ancient control mechanisms, thus explaining why a reversion to unicellular biology in cancer leads to these genes being involved in tumorigenesis.*

We moved the last comment here since it is closely linked with the first one. We deeply appreciate these insightful comments, which highlighted two important issues in the previous version.

First, we apologize that we did not explain the dating strategies well. The reason that *DDX11* or other genes could be dated as UC genes is due to the family-level

dating strategy or phylostratigraphy, which assigns the same age, that of the oldest founding member, to all homologous genes of the same gene family. In contrast, as in another line of practice including our own efforts [1-7], we performed a synteny-based dating strategy and thus differentiated ancestral and derived duplicates. In the updated version, we described the pros or cons of these two commonly used strategies and explained how we merged these two strategies to generate one unified age dataset. We additionally described that our synteny- based pipeline tends to be conservative (assigning genes older ages) compared to other synteny-based methods [2, 7].

Second, we feel embarrassed that we had not read the work mentioned by the referee [8], which proposed that UC genes confer adaptive mutability in cancer. We then performed a more thorough literature survey and gained a more comprehensive understanding of atavism. We thus agree with the referee that cell cycle related PSGs may represent UC gene derived duplicates and proved this hypothesis in the updated manuscript. We now interpreted the excess of these PSGs as the joint prediction of atavism and antagonistic pleiotropy. We accordingly revised the Abstract, Background, Results, Discussion and Methods while adding a few necessary citations including [8]. With this fundamental change, the conceptual framework of our manuscript has become more solid.

The new version reads as follows:

(1) Age dating related changes

# “Compilation of the gene age dataset and cancer omics datasets

To analyze the roles of PSGs in tumors, we first compiled a genome-wide gene age dataset spanning the most ancient UC genes to the youngest PSGs. Specifically, two strategies for dating gene origination have been developed: protein-family-based methods, also known as phylostratigraphy [22, 23, 47-49], and synteny-based methods [36, 37, 40, 50-53]. The former assigns the age of the founder gene to all homologous genes of the same gene family and thus does not differentiate between the child duplicates and the parental copy (Fig. 1a). In contrast, the latter distinguishes different duplicates on the basis of synteny (gene order) and parsimoniously infers the corresponding age. Thus, for genes harbored by the human genome, PSGs identified by the former strategy indicate that these genes

form primate-specific protein families (absent in non-primate species), while those genes identified with the latter strategy represent primate-specific loci.

**Fig. 1a** Schema of two strategies for dating gene ages. A phylogenetic tree covering four species is shown and the age inferences are marked along the tree. The purple and blue boxes mark the parental and child copies, respectively. The gray boxes indicate the genes around the focal gene while the white boxes indicate the absence of the child gene. The shaded region represents the syntenic alignment.

We compiled a single gene age dataset by merging these two dating strategies given their complementary nature (Methods). Since synteny degenerates faster than protein sequence during evolution, the synteny-based pipeline has been applied to date human genes within vertebrate evolution [40], while protein-level homology is detectable over a much longer period, thus, phylostratigraphy has been implemented to date ancient genes [22, 23]. We thus used the age data of group 9-14 after vertebrate split (Fig. 1b; Methods) generated via our previous synteny-based pipeline [40]. Genes predating the vertebrate split were classified into eight groups in a relatively new phylostratigraphy dataset [23]. We further divided all groups into 5236 UC genes, 7767 EM genes, 2704 mammal-specific (MM) genes and 423 PSGs (Fig. 1b, Additional file 1: Fig. S1a; Additional file 2: Table S1) and considered that UC and EM genes could serve as two controls in subsequent analyses given their respective roles in tumor promotion or repression [19-24].

Notably, the dataset of PSGs shows the well-known transcriptional and evolutionary features of young genes, *i.e.*, narrow expression [35, 54, 55] and fast evolution [56, 57]. First, almost half (190/423) of the PSGs showed tissue-biased expression (*e.g.*, testis bias, Additional file 1: Fig. S1b; Methods), while this

proportion declined in the three older age groups (43%-9%, Fig. 1b; Additional file 2: Table S1). Actually, due to pervasive incomplete duplication or relocation [58, 59], duplicate PSGs generally show narrower expression than their corresponding parental copies (Additional file 1: Fig. S1c). Second, duplicate PSGs tend to evolve faster than their older counterparts (Additional file 1: Fig. S1d) driven by positive selection or relaxation of functional constraint [56, 57, 60].” (Page 5-9)

“**Fig. S1c** Comparison of expression breadth between PSGs and their parental copies. Tissue number denotes the number of tissues in which the expression level (TPM) is higher than 1. **d** Comparison of evolution rate (*K*a/*K*s) between PSGs and the parental copies. For Panels c and d, one-sided Wilcoxon signed-rank test was implemented.”

Methods

# “Compilation of a unified gene age dataset

The gene age dataset was extracted from the synteny-based GenTree database [40], and further complemented with phylostratigraphy data generated in [23]. Although multiple phylostratigraphy datasets were generated in human [19-23], we chose [23] considering the relatively recent release time and data availability, and so as to reproduce the patterns (upregulation of UC genes and downregulation of EM genes) in [23].

As introduced in the Results, GenTree takes advantage of genome-wide synteny and performs gene-level dating parsimoniously along the vertebrate phylogenetic

tree, while phylostratigraphy performs family-level dating and can be used to infer the age of ancient genes when synteny become degenerated [40]. We excluded genes for which GenTree and phylostratigraphy produced conflicting age data. Genes from the phylostratigraphy dataset that could not be mapped to Ensembl v73 due to database version differences and genes that lacked age information in GenTree due to difficulty in inferring synteny [*e.g.*, genes situated in transposon- rich regions or assembly gaps in the outgroup species, [40]] were also discarded. We further divided the remaining genes postdating the vertebrate split in GenTree into 6 clades with at least 200 genes for each group (age group 9–14, Fig. 1b). For genes assigned to vertebrate common ancestors (the oldest age group in GenTree), we divided them into group 1–8 based on the phylostratigraphy analysis (Additional file 1: Fig. S1a). In addition, for synteny defined PSGs, we also analyzed their age distribution in the phylostratigraphy dataset [23] in order to test whether PSGs potentially involved in the cell cycle represent duplicates derived from gene families with UC genes as the founding members.

Note that our synteny-based dating strategy is conservative in terms of identification of young genes. That is, we previously performed extensive manual curation and found that we tend to assign genes to older ages and generated smaller young gene dataset compared to other synteny-based work in humans or flies [40, 53].

For each PSG, we downloaded our previously generated origination mechanism information [40]. In brief, we classified them as duplicate (with a paralog available) and potential *de novo* genes (without a paralog). The parental copy is defined as the most similar paralog with an age older than the focal PSG. The *K*a/*K*s values were also from the previous work [40].” (Page 30-31)

(2) The framework related changes Abstract

# “Background

Although being extensively studied, it remains unclear why humans bear a high risk of cancer. The antagonistic pleiotropy hypothesis predicts that primate-

specific genes (PSGs) tend to promote tumor, while the molecular atavism hypothesis predicts that PSGs involved in tumor may represent recently derived duplicates of unicellular (UC) genes. However, these predictions have not been tested.

# Results

By taking advantage of pan-cancer genomic data, we found the upregulation of PSGs across 13 cancer types, which is facilitated by copy number gain and promoter hypomethylation. Meta-analyses indicated that upregulated PSGs (uPSGs) tend to promote tumorigenesis and to play cell cycle related roles. The cell cycle related uPSGs predominantly represent derived duplicates of UC genes. We prioritized 15 uPSGs and performed an in-depth analysis of one UC gene derived duplicate involved in cell cycle, *i.e.*, *DDX11*. Genome-wide screening data and knockdown experiments demonstrated that *DDX11* is broadly essential across cancer cell lines. Importantly, non-neutral amino acid substitution patterns and increased expression indicate that *DDX11* has been subjected to positive selection. Finally, we found that cell cycle related uPSGs are also preferentially upregulated in the highly proliferative embryonic cerebrum (forebrain).

# Conclusions

Consistent with the predictions of the atavism and antagonistic pleiotropy hypotheses, PSGs, especially UC genes derived cell cycle related PSGs, contribute to the early proliferation of human cerebrum at the cost of hitchhiking by similarly proliferative cancer cells.” (Page 2-3)

Background

“Micro- and macroevolutionary practices could be combined to better understand cancer as in studies of molecular atavism and antagonistic pleiotropy. On the one hand, it was hypothesized decades ago that cancer cells are reminiscent of unicellular ancestors and oncogenesis emerges by reversing phylogeny [14, 15]. Nonetheless, this atavistic view was formally formulated more recently [16-18]. Phylostratigraphic or gene age analyses provide abundant supporting evidence:

(1) cancer related genes often emerge in unicellular (UC) ancestors or early

metazoan (EM) ancestors [19-22]; (2) UC genes tend to be upregulated in tumors, while EM genes are often downregulated [23, 24]. Therefore, atavism is increasingly accepted as a theoretical framework to understand cancer [4, 25-27] and adaptive mutability enabled by the ancient memory is even proposed as a target in tumor therapy [22, 28]. On the other hand, natural selection may maximize the fitness in youth at the cost of promoting diseases of aging [3, 29, 30]. Antagonism could be most intense for recent genetic changes due to lack of time resolving the negative pleiotropy [31, 32]. This hypothesis has rarely been tested at the genome-wide level with few exceptions, *e.g.*, one study has shown that human-specific enhancers underlie the cost of aging diseases including cancer [33].

Antagonistic pleiotropy predicts that recently originated new genes (novel gene loci emerging in recent evolution) [34, 35] should often contribute to tumor. Considering atavism and the fact that the majority of new genes are generated by DNA- or RNA-level duplication [36, 37], the link between tumors and new genes could be more pronounced for new genes as derived duplicates of UC genes. That is, primate-specific genes (PSGs, including human-specific genes) harbored by the human genome should fit these patterns. Studies including our own have already indicated tumor-promoting roles for a handful of PSGs, in addition to their normal function in fetal brain development or spermatogenesis [38-43].

Therefore, we analyzed whether PSGs promote tumor, whether PSGs involved in tumor represent UC gene derived duplicates and what normal functions of PSGs are hitchhiked by tumors, especially considering that most PSGs are poorly characterized [44, 45]. Specifically, by taking advantage of data generated by The Cancer Genome Atlas (TCGA) project [46], we demonstrated a pan-cancer global upregulation of PSGs, which was contributed by copy number gains and promoter hypomethylation. By integrating

functional data and evolutionary data, we showed that upregulated PSGs tend to facilitate tumor possibly due to their roles in the cell cycle and the majority of these cell cycle related PSGs are derived duplicates of UC genes. For one particularly strong case, *DDX11*, we corroborated its essentiality in cancer cells via knockdown assays and revealed

its fast evolution shaped by natural selection. We finally showed that upregulated

PSGs associated with the cell cycle, including *DDX11,* are also biasedly expressed during embryonic cerebral development, which involves extensive cell

proliferation, suggesting that similarly highly proliferative tumor recapitulated part of the cell cycle program that normally acts during this critical stage.” (Page 4-6)

Results

“Given the atavism hypothesis, the shared excess of cell cycle related genes in both uUC genes and uPSGs indicates the reactivation of dormant ancient memory. Thus, we expect that uPSGs potentially involved in the cell cycle should mainly represent derived duplicates of UC genes. Consistently, 67.6%-81.8% of cell cycle related uPSGs have been dated as UC genes in the protein-family-level age inference of phylostratigraphy, which is significantly higher than the overall background of PSGs (37.1%, *P* ≤ 0.003, Fig. 4c; Additional file 2: Table S4, Methods). ” (Page 18)

“Similar to the majority of cell cycle related uPSGs (Fig. 4c, Additional file 2: Table S4), *DDX11* represents a UC gene derived duplicate that emerged in recent evolution. ” (Page 22)

“Therefore, positive selection may drive the enhanced function of *DDX11* by modifying the critical protein domains and increasing its expression, which could be hitchhiked by the highly proliferative cell cycles of cancer. ” (Page 24)

“Since tumor hitchhiking of UC genes derived cell cycle related uPSGs (including *DDX11*) may represent antagonistic pleiotropy, we wondered which normal biological processes recruit these cell cycle related new genes under the force of positive selection. Considering that increased proliferation of neural progenitor or stem cells drives human cerebral expansion [85-88] and that cancer cells have been proposed to be similar to neural stem cells [*e.g.*, fast cell cycle or proliferation [89]], we hypothesized that these cell cycle related uPSGs emerge due to the selection in driving brain expansion. To this end, we analyzed whether a rapid proliferation stage of brain development recruits an excess of PSGs and whether this stage is a hot target of selection.” (Page 24)

“Altogether, the cell cycle program of embryonic cerebral development seems to be rewired by recruiting PSGs and modifying preexisting genes. ” (Page 26)

Discussion

“By integrating evolutionary and functional genomic data, we found that PSGs upregulated in tumors tend to be involved in cell cycle and that these cell cycle related genes mainly represent derived duplicates of UC genes. That is, we detected patterns compatible with the predictions of atavism and antagonistic pleiotropy. ” (Page 26)

“On the one hand, Darwinian medicine has championed the idea that disease susceptibility is predisposed by macroevolutionary history [1, 3, 4, 91]. Accordingly, studies of atavism and antagonistic pleiotropy provide clues for understanding tumor biology [22, 28, 33]. Herein, by building upon previous efforts [19-24], our meta-analyses across 13 cancer types demonstrated that the pan-cancer upregulation of UC genes involved in basic cellular machinery tends to promote tumor (Figs. 2c, 4a, Additional file 1: Fig. S4d) and thus corroborated the atavistic hypothesis. We identified a novel pattern in which PSGs especially those UC genes derived duplicates involved in cell cycle (*e.g.*, *DDX11*) show an analogous upregulation in tumors (Figs. 2c, 4b-c).” (Page 27)

“(3) since we focused on UC gene derived uPSGs involved in cell proliferation across multiple cancer types, PSGs recruited into other hallmarks (*e.g.*, immune invasion) or functional in specific cancer types warrant further investigation, especially considering PSGs’ biased expression in immune-related organs [Additional file 1: Fig. S1b, [40, 97]];” (Page 27-28)

“Interestingly, our analyses reassert the importance of the embryonic stage but showed a picture different from a few evolutionary changes proposed in the original hypothesis: the evolution of hundreds of genes appears to occur where multiple aspects of the cell cycle (*e.g.*, DNA replication/repair) are involved and coding changes or the origination of new genes occurs in addition to regulatory changes (Fig. 6). These results expanded upon the previous case studies where positive selection acts on genes related to DNA repair or spindle functions [*BRCA1* or *ASPM*, [110, 111]]. System-level rewiring occurs in the embryonic stage because the brain develops with the highest proliferation rate [112-114]. Dramatic proliferation induces cell cycle dysfunctions such as DNA damage and replication

stress in the brain [111, 115] and the majority of congenital abnormalities occur in this stage [85]. Thus, a specialized cell cycle program gets molded under positive selection where *DDX11*, *BRCA1* or *ASPM* could support accelerated proliferation more efficiently than their ancestral forms did. In parallel, tumors also face cell cycle dysfunction due to their similarly proliferative nature [78, 116], which necessitates the co-option of cell cycle related genes expressed in the embryonic cerebrum.

Notably, although the atavism and antagonistic pleiotropy hypotheses jointly predict that cell cycle related PSGs derived from UC genes are necessary to be hitchhiked by tumors, whether hitchhiking occurs or not depends on chance.” (Page 28-29)

“Overall, as atavism and antagonistic pleiotropy hypotheses predict, PSGs, especially those UC genes derived cell cycle related PSGs, contribute to the embryonic cerebral development at the cost of elevated tumor risk. Thus, our meta-analyses hint on both the upsides and downsides of human evolutionary heritage.” (Page 30)

Methods

“In addition, for synteny defined PSGs, we also analyzed their age distribution in the phylostratigraphy dataset [23] in order to test whether PSGs potentially involved in the cell cycle represent duplicates derived from gene families with UC genes as the founding members. ” (Page 31)

*There are several issues/questions surrounding some of the statistical analyses reported in the paper.*

Thanks for pointing out these issues and we addressed them as follows.

1. *Many of the analyses are only reported in the figure legends, and not mentioned in the Methods section, making it difficult to determine if the correct analysis has been applied.*

Thanks for highlighting this issue. We have now read through the whole manuscript to ensure that the methods underlying each figure are included in the Method section. Since Figs. 1-4 have been extensively revised, we explained how the Methods were modified in response to the specific comment (see below). For Figs. 5 and 6, we also confirmed whether the corresponding methods have been described.

2. *The authors need to specify both R version and package version for all R-based analyses as this is essential to ensure replicability of the analysis.*

We agree and added version numbers of R packages accordingly.

Methods

“All relevant R-based packages are summarized in Additional file 2: Table S10.” (Page 47)

A snapshot of Additional file 2: Table S10.

In addition, we also added version numbers for other software packages used in the analyses, *e.g.*, kallisto v0.43.1, STAR v2.6.1d, and Trimmomatic v0.39.

3. *Any models used in the survival analysis need to be specified in the methods. While all TCGA projects were built around primary tumor samples, some of the studies, such as the bladder cancer study used in the manuscript, included multiple primary, metastatic or recurrent tumors. It is unclear whether the data was either filtered to remove these tumors or whether a variable was included to account for this. Additionally, the survival model that can be inferred from the information given does not address well-known factors that would influence PFS like tumor stage, patient age, and patient sex. The survival analyses need to be re-run taking these characteristics into account, and it would be useful to report out the OR for each variable in the model along with the 95% CI. Also, as a detail for making sure survival is understood appropriately in the paper, it should always be referred to as progression-free survival, as most readers will see survival and assume overall survival as the end point. It would also be nice to see some of the survival curves in the supplementary data. You can use Xena to generate these for some of the PSG.*

Many thanks for these detailed and constructive comments, and we have followed all of them.

Regarding the TCGA samples, we previously only used primary tumors, and we have now clarified this issue in the Methods:

“We focused on primary tumor sample from 13 solid cancer types [marked by TCGA	barcodes	“01”, https://docs.gdc.cancer.gov/Encyclopedia/pages/TCGA_Barcode] and required tumor sample number not lower than 150 and normal sample number not lower than 15.” (Page 30)

We then re-implemented the survival analysis and revised our manuscript: (1) we explicitly specified Cox proportional hazards model and progression-free interval as end-point; (2) after integrating available clinical information (age/stage/gender) for each cancer type (Additional file 2: Table S3), we updated the results of survival analyses (Fig. 4a, Additional file 1: Fig. S4b) and recapitulate the previous patterns; (3) for each variable, we compiled the corresponding statistics in Cox model (false discovery rate or FDR along with its ranking information, hazard ratio together with 95% confidence intervals, available at GitHub due to its big file size); and (4) we added survival curves for two

representative upregulated PSGs (Additional file 1: Fig. S4a, S4c), *i.e.*, *TBC1D29*

passing the FDR cutoff and *DDX11* associated with a high rank (see below). The new version reads as follows:

Results

“First, similar to uUC genes, uPSGs tend to promote tumors as revealed by survival data analyses (Additional file 2: Table S3; Additional file 3; Methods). Specifically, we identified genes with their expression levels significantly (false discovery rate or FDR < 0.05) correlated with the progression-free interval (PFI) of patients.” (Page 14)

A snapshot of Additional file 2: Table S3.

A partial snapshot of Additional file 3.

“The results corroborated the tumor-promoting effect of uUC genes and tumor- inhibiting effects of dEM genes [20, 22, 23]. That is, the stronger expression of 31.7% and 1.9% of uUC genes was associated with unfavorable and favorable clinical outcomes, respectively (Fig. 4a). In contrast, the corresponding proportion becomes 11.3% and 11.7% for dEM genes, respectively. We further found that uPSGs were more similar to uUC genes in that higher expression levels of uPSGs were associated with shorter (26.8%) and longer (0.7%) PFIs, respectively. One such example is *TBC1D29* (FDR = 0.016, Additional file 1: Fig. S4a). In addition to this FDR-based analysis, we obtained similar patterns with the top 1500 genes showing the highest correlation with PFI (Additional file 1: Fig. S4b). *DDX11* serves as an example where its rank percentile reaches 0.6% (the 98th gene out of 16283 genes) despite its FDR of 0.18 (Additional file 1: Fig. S4c).” (Page 14-15)

**“Fig. S4a** The survival plot of *TBC1D29* in prostate adenocarcinoma (PRAD). **b** Stronger expression of uPSGs or uUC genes more often leads to unfavorable survival compared to that of dEM genes. This panel reproduces Fig. 4a by using the top 1500 genes most associated with the progression-free interval time. **c** The survival plot of *DDX11* in liver hepatocellular carcinoma (LIHC).**”**

Methods

“We implemented the Cox proportional hazards model with the survival package v2.43.3 in R to define prognostic genes as those whose expression levels were significantly associated with the survival time of patients after controlling for clinical variables (age/stage/gender) if applicable (Additional file 2: Table S3). Among multiple clinical endpoints, overall survival (OS) and PFI have been recommended for high-quality survival analyses [121]. Note that PFI data is

recommended for all 13 cancer types covered in this study, and OS data is problematic for three cancer types, where the endpoint needs a longer follow-up or the number of events is not sufficient. We thus used the PFI in subsequent analyses. We defined prognostic genes as those whose expression was significantly correlated with PFI (FDR < 0.05). ” (Page 36)

“Notably, in Additional file 3 and Additional file 1: Fig. S4a/c, we also showed the hazard ratio together with the 95% confidence interval. The hazard ratio is similar to the odds ratio and both of these values are often perceived as relative risk [137]. The major difference is that the hazard ratio is not constant while the odds ratio is. Therefore, the hazard ratio is more suitable for survival data analyses [137]. ” (Page 37)

Availability of data and materials

The R code for Figs. 1 to 6 and Additional file 3 are available at https://github.com/Zhanglab-IOZ/PSG-Tumor-Brain. (Page 47)

4. *Given that TCGA data is sample based, and samples should have gene expression data along with both copy number and methylation data, it would be more appropriate to correlate DNA copy number and/or percent methylation with gene expression for the data sets using the Spearman's correlation coefficient. This analysis will give a better idea of how much copy number and methylation contribute to expression difference than the current analysis. Additionally, the authors could do an all by all analysis across all tissues of origin and see if there are interesting relationships between genes that correspond to different age groups, for example: do you see interesting relationships between UC genes and the primate specific genes? The current analysis suggests association of amplification and hypomethylation with over-expression but does not support correlation strongly enough to justify causation. Doing the correlation analysis would demonstrate this point more forcefully. Since other factors beyond copy number and methylation status play a role in transcript abundance, reasonable correlation coefficients for self-self comparisons (e.g. gene 1 copy number and gene 1 expression) makes a much stronger argument for causation. This type of analysis also has the advantage of not*

*needing to classify genes as amplified or deleted which gets tricky in copy number data in tumors because the cut-offs used depend on the ploidy of the tumor.*

Again, thank you for these insightful comments. We previously also realized that “*classify genes as amplified or deleted which gets tricky in copy number data in tumors because the cut-offs used depend on the ploidy of the tumor*”. However, we did not consider the direct sample-level correlation analysis as proposed by the referee, which has been included in the revised manuscript. Moreover, we also appreciate the idea, *i.e.*, “*if there are interesting relationships between genes that correspond to different age groups*”. Based on this suggestion and on Comment 5 from Referee 2, we included UC and EM genes as controls whenever applicable and gained more insights into PSGs.

In the updated version, we replaced the previously problematic (*e.g.* cut-offs confounded by the ploidy) analysis with the sample-level analysis and included UC and EM genes.

Results

“Since gene expression in tumors is shaped by a myriad of factors including somatic copy number alterations (SCNAs), DNA methylation or histone modifications [24, 63, 64], we wondered which factors underlie the pan-cancer upregulation of PSGs. Considering that SCNA and promoter methylation are among the most often studied factors in gene deregulation in tumors [65, 66] and that these two types of data are available for the majority of TCGA samples (Fig. 1c), we analyzed their contribution by calculating how strong gene expression is correlated with copy number and promoter methylation, respectively.

We detected varying correlation intensity across gene age groups (Fig. 3): (1) consistent with [24], SCNA (and more specifically, amplification) is one major factor underlying the upregulation of UC genes with a median of 47.9% genes showing a strong correlation (Spearman *ρ* > 0.3, Methods) between expression level and copy number; (2) the extent of correlation between expression and SCNA is relatively moderate for EM genes, T-PSGs and B-PSGs with the proportions ranging between 2.9% and 20.0% and the pattern for methylation is similar (13.8%-21.5%); (3) amplification contributes much more toward upregulation of B-PSGs than to that of T-PSGs (20.0% *vs.* 2.9%); and (4) compared to amplification,

the decrease of methylation or hypomethylation is a stronger factor for the upregulation of T-PSGs (2.9% *vs.* 13.8%, respectively), which echoes the significance of methylation in regulating tissue-biased oncogenes [66]. We obtained similar results with a more stringent cutoff (*ρ* > 0.4, Additional file 1: Fig. S3c).

**Fig. 3** Pan-cancer proportion distribution of genes whose expression levels are strongly correlated with SCNA or promoter methylation. The Wilcoxon signed- rank test was used to measure the significance.

**Fig S3c** Pan-cancer proportion distribution of genes whose expression is significantly correlated with SCNA or promoter methylation. This panel follows the same convention as Fig. 3 except that a more stringent cutoff of 0.4/-0.4 was used (Methods).

Taken together, the correlation analyses indicate that the upregulation of PSGs is

contributed by amplification and promoter hypomethylation.” (Page 12-14)

Methods

“We implemented the Spearman correlation-based analysis to evaluate how strongly gene expression is affected by SCNA or promoter methylation. For each gene, we calculated the correlation between gene expression and SCNA/methylation within each cancer type. According to the genome-wide distribution of the correlation coefficient, 0.3/-0.3 was selected as the cutoff to define significantly correlated genes for SCNA/promoter methylation (Fig. 3). We also tested a more stringent threshold of 0.4/-0.4 and largely reproduced the patterns generated with the 0.3/-0.3 cutoff (Additional file 1: Fig. S3c).” (Page 35)

5. *It is unclear which analyses were controlled for multiple comparisons. The number of analyses reported guarantees more than one result is statistically significant due to chance alone. This needs to be addressed. The p.adjust function in the stats package offers several methods for doing the adjustment for multiple comparisons.*

Thanks. We followed the suggestion and performed multiple testing correction with FDR calculation where necessary. We accordingly revised the Results and Methods.

Results

“**Fig S1b** Distribution of tissue-biased PSGs. For each group, the enrichment fold was defined as the ratio of its proportion in PSGs divided by the overall genomic proportion (see also Methods). A binomial test was implemented, and only groups with more than 100 genes were included in this analysis. PSGs are overrepresented in testis/placenta/bone marrow biased genes and underrepresented in broadly transcribed genes or adult brain-biased genes. Both patterns are consistent with previous reports [9, 10].”

“**Fig. 2** Upregulation of PSGs in tumors. **a** Distribution of the median log2(Fold change) of 423 PSGs across tumors. Tumor types are sorted by decreasing values. **b** Distribution of the ssGSEA enrichment scores of PSGs across tumor types. For

each violin plot, the bar, the line and the curve indicate the interquartile range, the median and the probability density of the data, respectively. The median score of PSGs in normal samples was set as 0, *i.e.*, the normal median was subtracted from its counterpart in tumor samples. Tumors are sorted by decreasing scores. **c** Violin plots showing distributions of enrichment scores (median across tumors) of genes within 14 age groups. For each group (defined as in Fig. 1b), the median enrichment score was calculated separately for normal and tumor samples within each tumor type. Similar to Panel b, the median values in normal samples were set as 0. **d** Enrichment score-based expression patterns of broadly transcribed PSGs and tissue-biased PSGs across tumors. **e** Proportion comparison between pan- cancer upregulated and downregulated genes. One-sided Wilcoxon signed-rank test was used for panels a-d and one-sided binomial test was used for Panel e. Multiple testing correction was performed via false discovery rate (FDR).” (Page 13-14)

**“Fig. S2 Pan-cancer upregulation of PSGs. a-c** Distribution of enrichment scores: PSGs (**a**), 14 age groups (**b**), B-PSGs and T-PSGs (**c**). Only high-purity tumor samples are used in these three panels with figure conventions following Fig. 2b- d, respectively. **d** Proportion comparison between pan-cancer upregulated and downregulated genes. This panel follows the same convention as Fig. 2e except that a more stringent cutoff was used to define the differentially expressed genes (Methods).”

“**Fig. S4d-e** Enrichment of uPSGs, uUC genes and dEM genes in 50 annotated MSigDB hallmarks relative to the genomic background. For genes assigned to at least one MSigDB hallmark, we performed one-sided binomial test with multiple testing correction (FDR) to examine the distribution bias of these genes across hallmarks. Hallmarks were arranged and color-coded according to a total of eight corresponding functional categories. For the proliferation hallmark category, three cell cycle related hallmarks were labeled. For the other seven categories, one representative hallmark was labeled. Panel e follows the same convention except that genes with E2F binding motifs in the promoter region were removed to avoid the interference of this key transcription factor driving cell cycle.”

**“Fig. 6** Distribution of proportions of genes highly expressed in one stage of

cerebral development. Four series of proportions are shown: all genes and PSGs with biased expression in one stage, genes positively selected on coding and promoter regions. PCW, M and Y refer to postconceptional weeks, months and years, respectively. For each stage, we implemented binomial tests and examined whether PSGs and positively selected genes showed significant excess or depletion relative to the genomic background. Only four stages showed at least one significant (FDR < 0.1) test result and the top four GO terms for these stages are shown. For 19-24 PCW, no significant terms were identified.” (Page 26)

A partial snapshot of Additional file 2: Table S1.

A partial snapshot of Additional file 2: Table S8.

Methods

“Specifically, within each cancer type, we used the limma package v3.34.9 [127] to identify genes that were differentially expressed between tumor and normal samples with a false discovery rate (FDR) lower than 0.05.” (Page 35)

“We defined prognostic genes as those whose expression was significantly correlated with PFI (FDR < 0.05).” (Page 36)

# “Figures and statistical tests

We used violin plots to show the data distributions. The bar indicates the interquartile range (IQR) and the point indicates the median.

We estimated significance with Wilcoxon signed-rank tests, t-tests, Chi-square tests and binomial tests depending on the specific contexts. Unless otherwise specified, we implemented two-sided tests. Multiple test correction was performed via the FDR strategy as implemented in the p.adjust function in R v3.4.4.” (Page 46-47)

6. *In addition to the concerns about the details of the statistical analysis, some of the conclusions are too strong. For example, the conclusion that uPSGs "rewire" cell cycle is too strong. What can be concluded is that uPSGs play a role in proliferation and/or viability but no more because the assumption that correlated gene expression is indicative of common biological function is tenuous. To support their stated conclusions, the authors need to show that the partner genes are not potentially in a gene expression neighborhood. Gene expression neighborhoods are regions of the genome where genes have similar gene expression levels but are not involved in the same biological functions or pathways. One way to avoid including genes that are correlated by virtue of being in the same or similar gene expression neighborhoods is to be considerably stricter on the correlation coefficient filter. I would suggest that if correlated gene expression is how they want to map genes into known functions, they should use a cut-off of 0.7 or better, and then only pick partner genes that are on different chromosomes and are not regulated by E2F. E2F regulation, by virtue of*

*being essential to cell cycle control, will result in partner genes that map most abundantly to categories related to cell cycle control. They should also use DAVID and do a functional enrichment analysis to double check the results of any functional enrichment where they map their genes to the ontology by partner gene expression. The advantage of using DAVID is that it will do an enrichment across multiple ontologies simultaneously and cluster the results. Even if the genes are not well characterized, the authors should be able to tease out the biology underlying them and correlate that to their other analyses. This type of analysis will also mitigate the confounding effect of E2F control.*

We appreciate this set of insightful and constructive comments, and made changes accordingly. First, we have toned down those strong statements, especially the cell cycle related statements. Second, for MSigDB-based analyses, we largely recapitulated the overrepresentation of cell cycle related upregulated PSGs (Additional file 1: Fig. S4d-e, Methods) with chromosomal location or E2F partners controlled. Third, we replaced MSigDB as the DAVID annotation system and detected a similar excess of cell cycle related uPSGs (Additional file 1: Fig. S4f; Methods). Forth, together with Comments 2 and 3 of Referee 2, we acknowledged in the text that coexpression based analyses may cause false positives or false negatives. We, therefore, moved all correlation-based results to the supplemental file (Additional file 1: Fig. S4d-f), and kept only the results based on a curated high- quality cell cycle list in the main figure (Fig. 4b).

For the remaining one specific comment, *i.e.*, the correlation cutoff, we also followed it and found that this cutoff made the subsequent enrichment analysis impossible. That is, for coexpression related analyses, we used a correlation coefficient cutoff of 0.4 rather than 0.7 specified by the referee: with more stringent cutoffs such as 0.5 or higher, the number of coexpressed partner genes decreases dramatically (from a median of 249 to 24 or even lower) and makes the subsequent enrichment analyses unfeasible. We explained this issue in the Methods.

The new version of manuscript reads as follows:

Title:

# “Pan-cancer surveys indicate cell cycle related roles of primate-specific

**genes in tumors and embryonic cerebrum**” (Page 1) Abstract:

“Meta-analyses indicated that upregulated PSGs (uPSGs) tend to promote tumorigenesis and to play cell cycle related roles.” (Page 2)

Results:

# “Pan-cancer upregulated PSGs (uPSGs) tend to promote tumors and to play cell cycle related roles” (Page 14)

“Second, functional analyses indicate that uUC genes and uPSGs tend to be involved in cell cycle. Since PSGs are generally uncharacterized [44, 45], we could not perform the conventional functional enrichment analyses. Therefore, we mapped each gene to MSigDB annotated biological processes [67] on the basis of the fact that the expressions of genes involved in similar processes tend to be correlated [68]. As predicted by the atavism hypothesis [16-18], cell proliferation or metabolic hallmarks are often enriched in uUC genes while development or signaling hallmarks are overrepresented in dEM genes (Additional file 1: Fig. S4d- e; Methods). As for uPSGs, they were overrepresented in cell proliferation hallmarks, especially in three cell cycle related processes: mitotic spindle, G2/M checkpoint and E2F targets (Additional file 1: Fig. S4d-e). That is, 46 uPSGs may be involved in cell cycle (Additional file 2: Table S4). In addition to the cancer- oriented annotation system, *i.e*, MSigDB, we additionally used the general annotation system of DAVID [69] and confirmed the excess of cell cycle related uPSGs (Additional file 1: Fig. S4f; Additional file 2: Tables S4-S5; Methods).

**Fig. S4d-e** Enrichment of uPSGs, uUC genes and dEM genes in 50 annotated MSigDB hallmarks relative to the genomic background. For genes assigned to at least one MSigDB hallmark, we performed one-sided binomial test with multiple testing correction (FDR) to examine the distribution bias of these genes across hallmarks. Hallmarks were arranged and color-coded according to a total of eight corresponding functional categories. For the proliferation hallmark category, three cell cycle related hallmarks were labeled. For the other seven categories, one representative hallmark was labeled. Panel e follows the same convention except that genes with E2F binding motifs in the promoter region were removed to avoid the interference of this key transcription factor driving cell cycle. **f** The proportion of cell cycle related genes inferred via the of MSigDB, while other hallmarks refer to eight randomly sampled MSigDB gene sets (*e.g.*, apical junction; Methods). These two datasets serve as the positive and negative controls, respectively.. The Y-axis indicates the proportion of cell cycle related genes out of all genes assigned to at least one DAVID term.

A partial snapshot of Additional file 2: Table S4.

A partial snapshot of Additional file 2: Table S5.

Since correlation-based inference causes false positives or false negatives, we directly tested the enrichment of cell cycle functionality with a curated cell cycle gene list [70], which includes 666 genes involved in cell cycle progression, DNA replication or repair (Methods). We again found the overrepresentation of cell cycle genes in uPSGs or uUC genes and depletion of these genes in dEM genes, respectively (Fig. 4b). For example, 11 uPSGs play cell cycle related roles (Additional file 2: Table S4), which is overrepresented compared to the genomic proportion (binomial test *P* = 0.019).

**Fig.** 4**b** Proportion of genes covered by the 666-gene list.

Given the atavism hypothesis, the shared excess of cell cycle related genes in both uUC genes and uPSGs indicates the reactivation of dormant ancient memory. Thus, we expect that uPSGs potentially involved in the cell cycle should mainly represent derived duplicates of UC genes. Consistently, 67.6%-81.8% of cell cycle related uPSGs have been dated as UC genes in the protein-family-level age inference of phylostratigraphy, which is significantly higher than the overall background of PSGs (37.1%, *P* ≤ 0.003, Fig. 4c; Additional file 2: Table S4, Methods).” (Page17-18)

“Notably, both *DDX11* and *ZNF273* were identified by the MSigDB based correlation method and the 666-gene list to play cell cycle related roles, again suggesting the importance of the cell cycle program in tumors.

Altogether, an excess of UC genes derived uPSGs potentially related with the cell cycle are recruited into tumors.” (Page 19)

Discussions

“We further prioritized 15 upregulated PSGs potentially implicated in tumor, among which, *DDX11* has already been proposed to be a target for therapeutic interventions [75].” (Page 27)

Conclusions

“Thus, our meta-analyses hint on both the upsides and downsides of human evolutionary heritage.” (Page 30)

Methods

“In addition, for synteny defined PSGs, we also analyzed their age distribution in the phylostratigraphy dataset [23] in order to test whether PSGs potentially involved in the cell cycle represent duplicates derived from gene families with UC genes as the founding members.” (Page 31)

“To infer the functions of PSGs, we performed expression correlation-based enrichment analyses. For a given gene, we used the “ppcor” function in the R package psych v1.8.12 to calculate the Spearman correlation coefficient between its expression and that of other protein-coding genes while controlling for tumor purity within each cancer type. We took the median Spearman correlation coefficients across cancer types as a summary value for each gene pair (query and partner). To define the correlated partners, we retained genes with an absolute correlation coefficient above 0.4. With more stringent cutoffs such as 0.5 or beyond, the number of coexpressed partner genes decreases dramatically (from a median of 249 to 24 or even lower) and makes the subsequent enrichment analyses unfeasible. We further removed those partners that were located on the same chromosome as the given gene since genes located in chromosomal proximity may tend to be coexpressed regardless of their functions. We then downloaded 50 hallmark (biological processes) gene sets curated by MSigDB [67]. For each gene, we calculated the enrichment of its correlated genes across 50 hallmarks. We defined highly enriched hallmarks as those with statistical significance (odds ratio > 1, binomial test *P* < 0.05). With these steps, we assigned each query gene to zero or multiple significant hallmarks and tested whether the proportion of uPSGs assigned to one hallmark differed from that of the genomic background. For uUC and dEM genes, we performed similar enrichment analysis (Additional file 1: Fig. S4d). Since *E2Fs* are master regulators of the cell cycle and may regulate thousands of target genes [78, 81], we removed genes potentially regulated by E2Fs, *i.e.*, those with E2F binding sites in the promoter region annotated by MSigDB. A similar pattern was reproduced (Additional file 1: Fig. S4e).

In addition to MSigDB, we analogously mapped partner genes to DAVID functional terms [69] and reproduced similar patterns. Specifically, although DAVID is not a

cancer-oriented annotation system like MSigDB, it could perform enrichment analysis across multiple ontologies simultaneously and cluster the results. Since DAVID supports numerous ontologies, we chose all five default biological process or pathway oriented ontologies including "UP_KW_BIOLOGICAL_PROCESS", "GOTERM_BP_DIRECT", "BBID", "BIOCARTA" and "KEGG_PATHWAY". In

complement, we chose another two popular ontologies including "UP_KW_MOLECULAR_FUNCTION" and "GOTERM_MF_DIRECT". Clusters with enrichment scores not lower than 3 were considered and the most significant term within the cluster was retained. Different from MSigDB, which has three specific terms related to the cell cycle (mitotic spindle, G2/M checkpoint and E2F targets), we need to extract cell cycle related terms across seven ontologies in DAVID. To simplify this process, we searched G2/M checkpoint gene set of MSigDB in DAVID and identified 21 significant cell cycle related terms (*e.g.*, KW−0131~Cell cycle or GO:0005819~spindle, Additional file 2: Table S5). We also tested mitotic spindle or E2F target gene sets of MSigDB and retrieved a smaller subset of terms, which are largely covered by the previous 21 terms. We thus examined whether the coexpressed partner genes of a query gene were enriched in the 21 terms.

Different from MSigDB-based analyses, which were performed locally, we had to access the web server of DAVID to enable the enrichment analyses by uploading a list of partner genes for each individual query gene. Considering the limitation of website visit, we thus selected one positive control gene set (MSigDB mitotic spindle, G2/M checkpoint and E2F targets) including 552 genes and one negative control gene set including 804 genes. To represent the general scenario, the later list includes eight randomly sampled MSigDB gene sets, which covers all major categories of biological processes except cell proliferation and DNA damage (these two categories being related with cell cycle, Additional file 1: Fig. S4d): metabolic (bile acid metabolism), cellular component (apical junction), immune (complement, coagulation), pathway (protein secretion), signaling (androgen response, mTORC1 signaling) and development (epithelial-mesenchymal transition). We then used the RDAVIDWebService package v1.28.0 [138] to enable enrichment analyses for 1356 (552+804) times.

To validate the excess of cell cycle related PSGs based on MSigDB or DAVID, we further analyzed an independent cell cycle gene list, which is curated via a

literature survey and experimental analyses in normal cells [70]. This list includes 701 genes involved in a dozen distinct processes, such as cell cycle progression, DNA damage/replication or spindle assembly [70]. Despite of database version changes, the majority (666 or 95.0%) of these genes were covered in our analyses.” (Page 37-39)

7. *In Line 374, the conclusion is too strong. E2F likely coordinates DDX11 expression in conjunction with cell cycle, but DDX1, by virtue of being a helicase involved in S- phase, may have little to do with controlling cell cycle.*

Thanks. Although *DDX11* is involved in DNA replication or chromatid cohesion, it is indeed less likely to control the cell cycle. We thus toned down the writing as follows:

“Thus, under the coordination of *E2F*, *DDX11* interact with *Timeless* to support the tumor cell cycle.” (Page 22)

*REVIEWER #2*

*In this manuscript the authors explored the hypothesis of antagonistic pleiotropy by associating the gain of primate specific genes (PSGs) with cancer progression. They started with a phylostratification analysis and defined the birth time for each of the human genes. They observed generally upregulations of PGSs and UCs (unicellular ancestor genes), but downregulations of EMs (early metazoan genes), in cancers. The expression upregulations of PSGs in cancer can be ascribed mechanistically to copy number amplification or hypomethylation. Then, they showed the PSGs tend to be cell cycle related, and evolutionarily selected for human brain functions. In addition to reporting general patterns, a case gene (DDX11) was elaborated.*

Many thanks for this comprehensive and precise summary.

*I found the work overall very interesting. The analyses are well done and the presentation is generally clear, though there are a few issues that should be addressed before publication.*

We appreciate the referee’s enthusiasm and encouraging comments about our work. We have carefully read your comments and addressed them below.

1. *The first half of the paper is generally clear, but the second half, in particular, the brain part, is confusing. The authors should focus on PSGs, instead of brain biased genes (Fig. 6).*

We agree with this important comment and streamlined the manuscript accordingly. That is, we only kept the previous Fig. 6a as Fig. 6 in the main text, and the previous Fig. 6b becomes Additional file 1: Fig. S7b. These contents support the prediction of antagonistic pleiotropy that the rapid proliferation of embryonic brain recruits an excess of cell cycle related PSGs and this stage is a hot target of selection.

The new version reads as follows:

Results

# “Cell cycle related uPSGs tend to be involved in embryonic cerebral development and are subjected to positive selection

Since tumor hitchhiking of UC genes derived cell cycle related uPSGs (including *DDX11*) may represent antagonistic pleiotropy, we wondered which normal biological processes recruit these cell cycle related new genes under the force of positive selection. Considering that increased proliferation of neural progenitor or stem cells drives human cerebral expansion [85-88] and that cancer cells have been proposed to be similar to neural stem cells [*e.g.*, fast cell cycle or proliferation [89]], we hypothesized that these cell cycle related uPSGs emerge due to the selection in driving brain expansion. To this end, we analyzed whether a rapid proliferation stage of brain development recruits an excess of PSGs and whether this stage is a hot target of selection.

We reanalyzed a transcriptome dataset profiling embryonic development through the adult stages of seven organs [90]. To generate a global picture of gene expression in cerebrum (forebrain) development, we assigned genes toward one particular stage according to their biased expression (Additional file 1: Fig. S7a, Additional file 2: Table S7; Methods). We first reproduced previous observations [40, 57] (Fig. 6): (1) PSGs are disproportionately upregulated in the mid-fetal stages; (2) they are depleted in some postnatal samples in which genes involved in neuron ensheathment or synaptic transmission are disproportionately expressed. We then found that similar to the genome background the highest proportion of PSGs showed maximum expression in the embryonic stage (27.2%, Fig. 6). For this earliest stage, genes involved in the cell cycle are highly overrepresented with corresponding Gene Ontology (GO) terms (DNA replication/repair, cell division) contributing three out of the top four enriched terms (Additional file 2: Table S8). Consistently, we found the cell cycle related uPSGs (*e.g.*, *DDX11*) tended to be expressed in this stage regardless of whether the MSigDB correlation-based dataset (39.0%, one-sided binomial test *P* = 0.067) or the 666-gene list (80.0%, *P* = 0.0008) was used (Additional file 2: Table S4). Notably, the heightened activity of cell cycle genes was most pronounced in the cerebrum while the activity is significantly lower in other organs including cerebellum (Additional file 1: Fig. S7b).

**Fig. 6** Distribution of proportions of genes highly expressed in one stage of cerebral development. Four series of proportions are shown: all genes and PSGs with biased expression in one stage, genes positively selected on coding and promoter regions. PCW, M and Y refer to postconceptional weeks, months and years, respectively. For each stage, we implemented binomial tests and examined whether PSGs and positively selected genes showed significant excess or depletion relative to the genomic background. Only four stages showed at least one significant (FDR < 0.1) test result and the top four GO terms for these stages are shown. For 19-24 PCW, no significant terms were identified.

**Fig. S7 Additional analyses of genes expressed during the development. a** Genome-wide distribution of Z-scores. For a gene of interest, we defined a stage as the preferentially upregulated stage if this gene showed the highest expression in this stage and the Z-score was higher than 1.2 (top 10% percentile, see also

Methods). **b** ssGSEA raw expression score of cell cycle related genes across different organs in the embryonic stage**.** The 666-gene list was used to define cell cycle related genes.

Consistent with the positive selection signal of *DDX11* (Fig. 5e), we found that the embryonic stage was the only outlier with overrepresentation of genes with positive selection of their coding sequence or promoter regions (47 or 42.3%, 122 or 36.3%, Fig. 6; Additional file 2: Table S9; Methods). Consistent with the overall functional bias of embryonic genes, these 47 or 122 genes are also disproportionately related to the cell cycle (Additional file 2: Table S8).

Altogether, the cell cycle program of embryonic cerebral development seems to be rewired by recruiting PSGs and modifying preexisting genes. ” (Page 24-26)

Methods

# “Meta-analysis of genes with biased expression in the embryonic brain

For 4553 genes upregulated in the embryonic stage, we performed enrichment analysis with Metascape v3.5. Since the size limit of the input gene list was 3000, we thus randomly sampled 3000 genes 5 times and retained four GO terms that occurred at least 4 times, ranking in the top 5 in each sampling process (Additional file 2: Table S8). This is also why we chose the top 4 instead of the often used top 5 terms.

We downloaded the latest list of human protein-coding genes with signal of positive selection in the coding region of human lineage, which was based on the modified *K*a/*K*s test after correcting for GC content in primates [155]. Genes with promoter regions subjected to positive selection in human lineage were also compiled from [156] and only genes with *P*-values less than 0.05 were selected. Notably, numerous lists of noncoding regions under positive selection or human/primate-specific regulatory sequences have been published [108, 157- 161]. However, these lists were derived from functional genomics data of fetal or postnatal brains and thus could not be directly transferred to the embryonic brain. This is why we ultimately used the list generated in [156], which was computed based on the human genome directly and thus not anchored to a specific

developmental stage.

For all these meta-analyses, we used the Ensembl ID or gene symbol to cross- reference different datasets.” (Page 46)

2. *The cell cycle part is rather minor, and the evidence is indirect and weak. I feel it constitutes a major obstacle for an easy read. In particular, Fig. 4 is most confusing.*

Thank you for highlighting this important issue. As also suggested by Comment 6 of Referee 1, we revised the contents about the cell cycle from three aspects. First, we toned down those strong statements, especially the cell cycle related statements. Second, we largely recapitulated the overrepresentation of cell cycle related upregulated PSGs (Fig. 4b, Additional file 1: Fig. S4d-f, Methods) by following the suggestions of Referee 1 (controlling chromosomal location or E2F partners and analysis based on DAVID annotation system). Third, we moved all correlation-based results to the Additional file 1 (Fig. S4d-f) and kept only the results based on a curated cell cycle list in the main figure (Fig. 4b).

The new version of manuscript reads as follows:

Title:

# “Pan-cancer surveys indicate cell cycle related roles of primate-specific genes in tumors and embryonic cerebrum” (Page 1)

Abstract:

“Meta-analyses indicated that upregulated PSGs (uPSGs) tend to promote tumorigenesis and to play cell cycle related roles.” (Page 2)

Results:

# “Pan-cancer upregulated PSGs (uPSGs) tend to promote tumors and to play cell cycle related roles” (Page 14)

“Second, functional analyses indicate that uUC genes and uPSGs tend to be involved in cell cycle. Since PSGs are generally uncharacterized [44, 45], we could not perform the conventional functional enrichment analyses. Therefore, we mapped each gene to MSigDB annotated biological processes [67] on the basis of

the fact that the expressions of genes involved in similar processes tend to be correlated [68]. As predicted by the atavism hypothesis [16-18], cell proliferation or metabolic hallmarks are often enriched in uUC genes while development or signaling hallmarks are overrepresented in dEM genes (Additional file 1: Fig. S4d- e; Methods). As for uPSGs, they were overrepresented in cell proliferation hallmarks, especially in three cell cycle related processes: mitotic spindle, G2/M checkpoint and E2F targets (Additional file 1: Fig. S4d-e). That is, 46 uPSGs may be involved in cell cycle (Additional file 2: Table S4). In addition to the cancer- oriented annotation system, *i.e*, MSigDB, we additionally used the general annotation system of DAVID [69] and confirmed the excess of cell cycle related uPSGs (Additional file 1: Fig. S4f; Additional file 2: Tables S4-S5; Methods).

**Fig. S4d-e** Enrichment of uPSGs, uUC genes and dEM genes in 50 annotated MSigDB hallmarks relative to the genomic background. For genes assigned to at least one MSigDB hallmark, we performed one-sided binomial test with multiple testing correction (FDR) to examine the distribution bias of these genes across hallmarks. Hallmarks were arranged and color-coded according to a total of eight corresponding functional categories. For the proliferation hallmark category, three

cell cycle related hallmarks were labeled. For the other seven categories, one representative hallmark was labeled. Panel e follows the same convention except that genes with E2F binding motifs in the promoter region were removed to avoid the interference of this key transcription factor driving cell cycle. **f** The proportion of cell cycle related genes inferred via the of MSigDB, while other hallmarks refer to eight randomly sampled MSigDB gene sets (*e.g.*, apical junction; Methods). These two datasets serve as the positive and negative controls, respectively.. The Y-axis indicates the proportion of cell cycle related genes out of all genes assigned to at least one DAVID term.

A partial snapshot of Additional file 2: Table S4.

A partial snapshot of Additional file 2: Table S5.

Since correlation-based inference causes false positives or false negatives, we directly tested the enrichment of cell cycle functionality with a curated cell cycle gene list [70], which includes 666 genes involved in cell cycle progression, DNA replication or repair (Methods). We again found the overrepresentation of cell

cycle genes in uPSGs or uUC genes and depletion of these genes in dEM genes, respectively (Fig. 4b). For example, 11 uPSGs play cell cycle related roles (Additional file 2: Table S4), which is overrepresented compared to the genomic proportion (binomial test *P* = 0.019).

**Fig.** 4**b** Proportion of genes covered by the 666-gene list.

Given the atavism hypothesis, the shared excess of cell cycle related genes in both uUC genes and uPSGs indicates the reactivation of dormant ancient memory. Thus, we expect that uPSGs potentially involved in the cell cycle should mainly represent derived duplicates of UC genes. Consistently, 67.6%-81.8% of cell cycle related uPSGs have been dated as UC genes in the protein-family-level age inference of phylostratigraphy, which is significantly higher than the overall background of PSGs (37.1%, *P* ≤ 0.003, Fig. 4c; Additional file 2: Table S4, Methods).” (Page17-18)

“Notably, both *DDX11* and *ZNF273* were identified by the MSigDB based correlation method and the 666-gene list to play cell cycle related roles, again suggesting the importance of the cell cycle program in tumors.

Altogether, an excess of UC genes derived uPSGs potentially related with the cell cycle are recruited into tumors.” (Page 19)

Discussions

“We further prioritized 15 upregulated PSGs potentially implicated in tumor,

among which, *DDX11* has already been proposed to be a target for therapeutic interventions [75].” (Page 27)

Conclusions

“Thus, our meta-analyses hint on both the upsides and downsides of human evolutionary heritage.” (Page 30)

Methods

“In addition, for synteny defined PSGs, we also analyzed their age distribution in the phylostratigraphy dataset [23] in order to test whether PSGs potentially involved in the cell cycle represent duplicates derived from gene families with UC genes as the founding members.” (Page 31)

“To infer the functions of PSGs, we performed expression correlation-based enrichment analyses. For a given gene, we used the “ppcor” function in the R package psych v1.8.12 to calculate the Spearman correlation coefficient between its expression and that of other protein-coding genes while controlling for tumor purity within each cancer type. We took the median Spearman correlation coefficients across cancer types as a summary value for each gene pair (query and partner). To define the correlated partners, we retained genes with an absolute correlation coefficient above 0.4. With more stringent cutoffs such as 0.5 or beyond, the number of coexpressed partner genes decreases dramatically (from a median of 249 to 24 or even lower) and makes the subsequent enrichment analyses unfeasible. We further removed those partners that were located on the same chromosome as the given gene since genes located in chromosomal proximity may tend to be coexpressed regardless of their functions. We then downloaded 50 hallmark (biological processes) gene sets curated by MSigDB [67]. For each gene, we calculated the enrichment of its correlated genes across 50 hallmarks. We defined highly enriched hallmarks as those with statistical significance (odds ratio > 1, binomial test *P* < 0.05). With these steps, we assigned each query gene to zero or multiple significant hallmarks and tested whether the proportion of uPSGs assigned to one hallmark differed from that of the genomic background. For uUC and dEM genes, we performed similar enrichment analysis (Additional file 1: Fig. S4d). Since *E2Fs* are master regulators of the cell cycle and may regulate thousands of target genes [78, 81], we removed genes potentially

regulated by E2Fs, *i.e.*, those with E2F binding sites in the promoter region annotated by MSigDB. A similar pattern was reproduced (Additional file 1: Fig. S4e).

In addition to MSigDB, we analogously mapped partner genes to DAVID functional terms [69] and reproduced similar patterns. Specifically, although DAVID is not a cancer-oriented annotation system like MSigDB, it could perform enrichment analysis across multiple ontologies simultaneously and cluster the results. Since DAVID supports numerous ontologies, we chose all five default biological process or pathway oriented ontologies including "UP_KW_BIOLOGICAL_PROCESS", "GOTERM_BP_DIRECT", "BBID", "BIOCARTA" and "KEGG_PATHWAY". In

complement, we chose another two popular ontologies including "UP_KW_MOLECULAR_FUNCTION" and "GOTERM_MF_DIRECT". Clusters with enrichment scores not lower than 3 were considered and the most significant term within the cluster was retained. Different from MSigDB, which has three specific terms related to the cell cycle (mitotic spindle, G2/M checkpoint and E2F targets), we need to extract cell cycle related terms across seven ontologies in DAVID. To simplify this process, we searched G2/M checkpoint gene set of MSigDB in DAVID and identified 21 significant cell cycle related terms (*e.g.*, KW−0131~Cell cycle or GO:0005819~spindle, Additional file 2: Table S5). We also tested mitotic spindle or E2F target gene sets of MSigDB and retrieved a smaller subset of terms, which are largely covered by the previous 21 terms. We thus examined whether the coexpressed partner genes of a query gene were enriched in the 21 terms.

Different from MSigDB-based analyses, which were performed locally, we had to access the web server of DAVID to enable the enrichment analyses by uploading a list of partner genes for each individual query gene. Considering the limitation of website visit, we thus selected one positive control gene set (MSigDB mitotic spindle, G2/M checkpoint and E2F targets) including 552 genes and one negative control gene set including 804 genes. To represent the general scenario, the later list includes eight randomly sampled MSigDB gene sets, which covers all major categories of biological processes except cell proliferation and DNA damage (these two categories being related with cell cycle, Additional file 1: Fig. S4d): metabolic (bile acid metabolism), cellular component (apical junction), immune (complement, coagulation), pathway (protein secretion), signaling (androgen

response, mTORC1 signaling) and development (epithelial-mesenchymal transition). We then used the RDAVIDWebService package v1.28.0 [138] to enable enrichment analyses for 1356 (552+804) times.

To validate the excess of cell cycle related PSGs based on MSigDB or DAVID, we further analyzed an independent cell cycle gene list, which is curated via a literature survey and experimental analyses in normal cells [70]. This list includes 701 genes involved in a dozen distinct processes, such as cell cycle progression, DNA damage/replication or spindle assembly [70]. Despite of database version changes, the majority (666 or 95.0%) of these genes were covered in our analyses.” (Page 37-39)

3. *The authors conducted a lot of analyses, some of which are not obviously necessary. In addition, too many details are described in main text. The authors shall make full use of the supplementary part and the method part to make the paper concise and fluent.*

We agree with this comment and streamlined the manuscript accordingly. On the one hand, we deleted or moved less important contents to the supplementary figure: (1) for Fig. 3, we deleted the previous contents and presented only the more decisive contents by following Comment 4 of Referee 1; (2) for Fig. 4, as in the response to Comment 2, we presented only the panel based on a curated cell cycle gene list in the main figure; (3) for Fig. 5, we moved less important Panel b and f to Additional file 1: Fig. S5; and (4) for Fig. 6, as in the response to Comment 1, we significantly shortened the brain-related contents. On the other hand, we kept only the relatively more important methodological details in the Results section. For example, the methods of survival analyses in the Results were previously described as follows:

“Specifically, we performed regression analysis within each cancer type by correlating gene expression level and survival time of patients while accounting for tumor purity (Methods). Based on the number of cancer types for which gene expression was significantly (false discovery rate FDR<0.05) correlated with survival, we divided genes into four groups (Supplemental Table S3): favorable where higher expression was more often correlated with longer survival;

unfavorable where higher expression was more often correlated with shorter survival; intermediate if the number of cancer types was the same in both directions; non-prognostic where there was no significant regression signal across all cancer types.”

We have shortened this section as follows:

“Specifically, we identified genes with their expression levels significantly (false discovery rate or FDR < 0.05) correlated with the progression-free interval (PFI) of patients. We then divided genes into favorable group, unfavorable group, intermediate group and non-prognostic group on the basis of the number of cancer types for which their higher expression was correlated with longer or shorter PFI.” (Page 14)

4. *It's ok to state that, according to antagonistic pleiotropy, PSGs may promote cancer. But, given there are both positive and negative regulations in gene networks, why PSGs have to be upregulated to promote cancer?*

Many thanks for this insightful comment. We focused on upregulated PSGs due to the following reason: we used UC and EM genes, which are up- and downregulated in tumors, respectively, as controls; thus, we took upregulation as a signal to detect PSGs that are more likely to be involved in tumors. However, we do acknowledge the possibility that repression of some PSGs may promote tumor, too.

We clarified this issue in both Discussion and Methods section:

Discussion

“and (4) we herein focused on PSGs upregulated in tumors, but it is possible that the down-regulation of some PSGs might also contribute to tumor.” (Page 28)

Methods

“Note that we used upregulation to enrich PSGs more likely involved in tumors since genes beneficial for tumors (*e.g.*, UC genes) tend to be upregulated in tumors. In contrast, other PSGs especially those downregulated PSGs may repress tumors as did by genes emerging in early metazoan evolution (EM genes).” (Page 33)

5. *The Fig. 2c and 2e are most interesting. Ideally, the functional characterizations of PSGs in the later parts should be accompanied by the same characterizations of the UC and EM genes.*

We deeply appreciate this important comment. As aforementioned, the UC and EM genes serve as two natural controls of PSGs. We have added these two control gene lists to Fig. 3, Additional file 1: Fig. S3, Fig. 4 and Additional file 1: Fig. S4 whenever applicable. By recapitulating previous findings related to UC and EM genes, the updated version corroborated the functionality of PSGs and renders the logical flow much stronger.

The new version reads as follows:

# “Amplification and promoter hypomethylation contribute to the upregulation of PSGs

PSGs’ higher tumor expression might reflect their lower expression in normal samples, and thus they could therefore be more easily upregulated in tumors. Indeed, both B- and T-PSGs upregulated in tumors tended to have lower expression in normal controls compared to those downregulated in tumors (Additional file 1: Fig. S3a). However, UC and EM genes showed an analogous pattern. Moreover, although B-PSGs are more highly expressed than T-PSGs in normal samples, both showed a similar extent of upregulation in tumors (Additional file 1: Figs. S3a-b). Therefore, the upregulation of PSGs in tumors cannot be solely attributed to their lower expression level in normal samples.” (Page 12)

“**Fig. S3 Factors underlying up- or downregulation of genes in tumors. a** Comparison of normal expression levels between genes up- and downregulated in tumors. **b** Comparison of pan-cancer upregulated gene proportion. **c** Pan-cancer proportion distribution of genes whose expression is significantly correlated with SCNA or promoter methylation. This panel follows the same convention as Fig. 3 except that a more stringent cutoff of 0.4/-0.4 was used (Methods).”

“We detected varying correlation intensity across gene age groups (Fig. 3): (1) consistent with [24], SCNA (and more specifically, amplification) is one major factor underlying the upregulation of UC genes with a median of 47.9% genes showing a strong correlation (Spearman *ρ* > 0.3, Methods) between expression level and copy number; (2) the extent of correlation between expression and SCNA is relatively moderate for EM genes, T-PSGs and B-PSGs with the proportions ranging between 2.9% and 20.0% and the pattern for methylation is similar (13.8%-21.5%); (3) amplification contributes much more toward upregulation of B-PSGs than to that of T-PSGs (20.0% *vs.* 2.9%); and (4) compared to amplification, the decrease of methylation or hypomethylation is a stronger factor for the upregulation of T-PSGs (2.9% *vs.* 13.8%, respectively), which echoes the significance of methylation in regulating tissue-biased oncogenes [66]. We

obtained similar results with a more stringent cutoff (*ρ* > 0.4, Additional file 1: Fig. S3c). ” (Page 13)

“**Fig. 3** Pan-cancer proportion distribution of genes whose expression levels are strongly correlated with SCNA or promoter methylation. The Wilcoxon signed- rank test was used to measure the significance.”

# “Pan-cancer upregulated PSGs (uPSGs) tend to promote tumors and to play cell cycle related roles

We next analyzed whether and how tumors benefit from upregulated PSGs by integrating survival data, pathway enrichment data and gene essentiality data in cancer cell lines (Methods). To identify more important genes, we focused only on the pan-cancer up- or downregulated genes (Fig. 2e) including upregulated UC genes (uUC genes), downregulated EM genes (dEM genes) and uPSGs. ” (Page 14)

“Second, functional analyses indicate that uUC genes and uPSGs tend to be involved in cell cycle. Since PSGs are generally uncharacterized [44, 45], we could not perform the conventional functional enrichment analyses. Therefore, we mapped each gene to MSigDB annotated biological processes [67] on the basis of the fact that the expressions of genes involved in similar processes tend to be correlated [68]. As predicted by the atavism hypothesis [16-18], cell proliferation or metabolic hallmarks are often enriched in uUC genes while development or signaling hallmarks are overrepresented in dEM genes (Additional file 1: Fig. S4d- e; Methods). As for uPSGs, they were overrepresented in cell proliferation hallmarks, especially in three cell cycle related processes: mitotic spindle, G2/M

checkpoint and E2F targets (Additional file 1: Fig. S4d-e). That is, 46 uPSGs may be involved in cell cycle (Additional file 2: Table S4). In addition to the cancer- oriented annotation system, *i.e*, MSigDB, we additionally used the general annotation system of DAVID [69] and confirmed the excess of cell cycle related uPSGs (Additional file 1: Fig. S4f; Additional file 2: Tables S4-S5; Methods).

**Fig. S4d-e** Enrichment of uPSGs, uUC genes and dEM genes in 50 annotated MSigDB hallmarks relative to the genomic background. For genes assigned to at least one MSigDB hallmark, we performed one-sided binomial test with multiple testing correction (FDR) to examine the distribution bias of these genes across hallmarks. Hallmarks were arranged and color-coded according to a total of eight corresponding functional categories. For the proliferation hallmark category, three cell cycle related hallmarks were labeled. For the other seven categories, one representative hallmark was labeled. Panel e follows the same convention except that genes with E2F binding motifs in the promoter region were removed to avoid the interference of this key transcription factor driving cell cycle.

Since correlation-based inference causes false positives or false negatives, we directly tested the enrichment of cell cycle functionality with a curated cell cycle gene list [70], which includes 666 genes involved in cell cycle progression, DNA replication or repair (Methods). We again found the overrepresentation of cell cycle genes in uPSGs or uUC genes and depletion of these genes in dEM genes, respectively (Fig. 4b). For example, 11 uPSGs play cell cycle related roles (Additional file 2: Table S4), which is overrepresented compared to the genomic

proportion (binomial test *P* = 0.019).” (Page 17-18)

“**Fig.** 4**b** Proportion of genes covered by the 666-gene list.”

6. *Do absolute expression levels underlie the observation of up- or down-regulations. For example, lowly expressed genes cannot be down-regulated further, and highly expressed genes tend to show expression reduction.*

We appreciate this insightful comment. We found that the up-/down-regulation of genes could be affected by their expression level in normal samples but the upregulation bias of PSGs could be only partially attributed to this bias.

The new version reads as follows:

“Indeed, both B- and T-PSGs upregulated in tumors tended to have lower expression in normal controls compared to those downregulated in tumors (Additional file 1: Fig. S3a). However, UC and EM genes showed an analogous pattern. Moreover, although B-PSGs are more highly expressed than T-PSGs in normal samples, both showed a similar extent of upregulation in tumors (Additional file 1: Figs. S3a-b). Therefore, the upregulation of PSGs in tumors cannot be solely attributed to their lower expression level in normal samples.” (Page 12)

“**Fig. S3a** Comparison of normal expression levels between genes up- and downregulated in tumors. **b** Comparison of pan-cancer upregulated gene proportion.”

7. *Why is the grey bar wider in Fig. 6a?*

We are sorry for this confusion. Previously, we widened the genomic background group (gray bar) to make the comparison more visible; in the revision, we’ve made all bars equally wide to avoid such misunderstanding. Please see our response to Comment 1 for the updated Fig. 6.

References

1. Bai YS, Casola C, Feschotte C, Betran E: **Comparative genomics reveals a constant rate of origination and convergent acquisition of functional retrogenes in Drosophila.** *Genome Biology* 2007, **8**.
2. Shao Y, Chen C, Shen H, He BZ, Yu D, Jiang S, Zhao S, Gao Z, Zhu Z, Chen X, et al: **GenTree, an integrated resource for analyzing the evolution and function of primate-specific coding genes.** *Genome Res* 2019, **29:**682-696.
3. Potrzebowski L, Vinckenbosch N, Marques AC, Chalmel F, Jegou B, Kaessmann H: **Chromosomal gene movements reflect the recent origin and biology of therian sex chromosomes.** *Plos Biology* 2008, **6:**709-716.
4. Zhang YE, Vibranovski MD, Krinsky BH, Long MY: **Age-dependent chromosomal distribution of male-biased genes in Drosophila.** *Genome Research* 2010, **20:**1526-1533.
5. Zhang YE, Vibranovski MD, Landback P, Marais GAB, Long M: **Chromosomal**

# Redistribution of Male-Biased Genes in Mammalian Evolution with Two Bursts of Gene Gain on the X Chromosome. *PLoS Biol* 2010, 8:e1000494.

6. Zhou Q, Zhang GJ, Zhang Y, Xu SY, Zhao RP, Zhan ZB, Li X, Ding Y, Yang SA, Wang W: **On the origin of new genes in Drosophila.** *Genome Research* 2008, **18:**1446-1455.
7. Xia S, VanKuren NW, Chen C, Zhang L, Kemkemer C, Shao Y, Jia H, Lee U, Advani AS, Gschwend A, et al: **Genomic analyses of new genes and their phenotypic effects reveal rapid evolution of essential functions in Drosophila development.** *PLoS Genet* 2021, **17:**e1009654.
8. Cisneros L, Bussey KJ, Orr AJ, Miočević M, Lineweaver CH, Davies P: **Ancient genes establish stress-induced mutation as a hallmark of cancer.** *PloS one* 2017, **12:**e0176258.

**Second round of review**

**Reviewer 2**

The authors have satisfactorily addressed my concerns. I have no further comments.
